# Upregulation of CD90 correlates with T lineage commitment at the DN2a stage of T cell development

**DOI:** 10.3389/fimmu.2026.1940310

**Published:** 2026-09-17

**Authors:** Dhruti Parikh, Karen Gu, Sara Tomei, Tom S. Weber, Shalin H. Naik, Mark M. W. Chong

**Affiliations:** 1 St Vincent’s Institute of Medical Research, Fitzroy, VIC, Australia; 2 Department of Medicine (St Vincent’s), University of Melbourne, Fitzroy, VIC, Australia; 3 Walter and Eliza Hall Institute of Medical Research, Parkville, VIC, Australia; 4 Department of Medical Biology, University of Melbourne, Parkville, VIC, Australia

**Keywords:** cell surface markers, CITE-seq, lineage commitment, RNA-Seq, T cell development, thymus

## Abstract

Early thymic progenitors (ETPs) retain the potential for alternative fates but commit to the T-cell lineage as they progress through the CD4^−^CD8^−^ double-negative (DN) thymocyte stages early in T-cell development. Current evidence suggests that T-lineage commitment occurs during the transition from the DN2a to DN2b stage, a process characterized by downregulation of c-KIT and CD44 expression. Moreover, Bcl11b activation in DN2a thymocytes closely coincides with T-lineage commitment. Despite this heterogeneity in Bcl11b-YFP expression within DN2a thymocytes, these cells have predominantly been studied as a single population due to the absence of cell-surface markers capable of resolving the corresponding substages. To define the T-lineage commitment transition process at higher resolution, we profiled c-KIT-expressing DN thymocytes in the mouse thymus using cellular indexing of transcriptomes and epitopes by sequencing (CITE-seq). This revealed heterogeneity within DN2a thymocytes. We identified two subpopulations, which we termed DN2a-1 and DN2a-2, based on differences in CD34, CD90, and *Bcl11b* expression. DN2a-1 thymocytes co-expressed multipotency and T-lineage-associated markers, whereas DN2a-2 cells mainly displayed a transcriptional profile consistent with T-lineage commitment. Functional assays demonstrated that DN2a-1 thymocytes were more developmentally advanced than ETPs but remained multipotent, whereas DN2a-2 thymocytes were more mature and largely restricted to the T lineage. Furthermore, *in vivo* clonal lineage tracing indicated that these subsets were on a developmental continuum. Together, these findings reveal that the heterogeneous DN2a stage can be resolved further by CD90 expression into pre- and post-T lineage-restricted subsets.

## Introduction

T cells develop from hematopoietic stem and progenitor cells (HSPCs) that migrate from the bone marrow to the thymus, where the unique thymic microenvironment drives the initiation and progression of the T-cell developmental program (1). During the early stages of thymopoiesis, thymocytes progressively lose the potential to adopt alternative fates and become committed to the T-lineage. Although key regulators of T-lineage specification and commitment are well defined (1), a deeper understanding of the developmental transition associated with T-lineage commitment is needed to accurately delineate the transcriptional and epigenetic mechanisms that establish and maintain T-cell identity.

The earliest stages of thymic T-cell development are collectively termed the DN stages because thymocytes lack expression of the CD4 and CD8 coreceptors (2). In mice, ETPs, a c-KIT^hi^ subpopulation of DN1 (CD44^+^CD25^-^) stage, represent the earliest and least differentiated stage of thymic T-cell development (2). ETPs co-express HSPC- and T-lineage-associated genes (3), reflecting their multilineage potential, including the ability to differentiate into T cells, B cells, myeloid cells, dendritic cells (DCs), and innate lymphoid cells (ILCs) (4–7). B lineage potential is lost at the subsequent DN2a (c-KIT^hi^CD44^+^CD25^+^) stage (5, 6, 8), whereas access to all non-T lineages is lost entirely by the DN2b (c-KIT^lo^CD44^+^CD25^+^) stage (9, 10), coincident with active repression of HSPC-associated genes (1, 10). Accordingly, T-lineage commitment is thought to occur during the DN2a-to-DN2b transition. Additional cell-surface markers, including CD90 (Thy1), FLT3, CD24, and the IL-7 receptor (IL-7R), along with reporter mouse models, have further refined the delineation of these developmental stages (1, 11, 12).

Expression of HSPC-associated transcription factors, such as *Spi1* (encoding PU.1) and *Bcl11a*, is proposed to decelerate T-lineage progression (13, 14). In contrast, Notch signaling and expression of key T-lineage transcription factors TCF1, GATA3, RUNX1, IKAROS, and E proteins drive the global regulatory changes in gene expression and chromatin accessibility required to promote T-lineage specification and commitment (1, 15). Importantly, activation of the transcription factor BCL11B coincides closely with functional T-lineage commitment and can be used to demarcate multipotent and T-lineage-committed DN2a thymocytes (11). BCL11B-deficient DN thymocytes are arrested at the DN2 stage and resemble immature thymocytes that have increased capacity to differentiate into natural killer (NK) cells (16–18). Although *Bcl11b* reporter mice remain invaluable for defining the transition to T-lineage commitment at the DN2a stage (11), prospective identification of DN2a substages using cell-surface markers remains poorly defined. Therefore, many studies still assess DN2a thymocytes as a single population (19, 20). Single-cell technologies provide the resolution to capture cellular heterogeneity and define developmental transitions through the simultaneous profiling of multiple molecular modalities. However, recent single-cell studies of early thymopoiesis have largely focused on ETPs (3, 21–23), leaving the cellular and molecular landscape of subsequent stages less well defined. Fine-mapping of the DN2a substage at the single-cell level, particularly with respect to cell-surface protein expression, is therefore required. Furthermore, the current knowledge of the T-lineage commitment transition is largely derived from *in vitro* systems, highlighting the need for more refined *in vivo* analyses.

Here, we used CITE-seq to profile gene expression and cell-surface protein abundance simultaneously. We identified differential expression of CD90 and CD34 as markers that can resolve DN2a thymocytes that remain multipotent from those that are T-lineage restricted. Using these markers, we defined the developmental kinetics and lineage potential of distinguishable DN2a substages *in vitro* and *in vivo*. Together, these findings provide a refined framework for dissecting DN2a heterogeneity and establish a more precise map of the molecular and cellular changes associated with T-lineage commitment in the postnatal mouse thymus.

## Materials and methods

### Mice

*Rag1*^−/−^ (24), *Spi1^fl/fl^* (25), *Spib*^−/−^ (26), *Spi1^gfp^* (27), *Rosa26*-CreER^T2^ (28), *Spib^tdTomato^* (29), *Irf8^gfp/+^* (30), and LoxCode (31, 32) mice have been described previously. All animals were analyzed at 4 to 8 weeks of age, unless indicated otherwise.

*Spi1^fl/fl^* and *Spi1^fl/fl^Rosa26*-CreER^T2^ mice were treated with 5 mg of 4-hydroxytamoxifen (4-OHT) by oral gavage at 17 and 21 days of age, and the thymi were harvested at 24 days of age. LoxCode/*Rosa26*-CreER^T2^ mice were injected intravenously with 100 μg of 4-OHT (Sigma-Aldrich) in KolliPhor at 3 weeks of age, and the thymus was harvested at 5 weeks of age.

### Primary cell purification and processing

Thymi were dissociated through a metal sieve to obtain single-cell suspensions, then filtered through a 70 μm cell strainer. The suspensions were depleted with anti-CD4 and anti-CD8α MACS MicroBeads (Miltenyi Biotec) to enrich for DN thymocytes for sorting.

When analyzing both T and non-T cells, the thymus or spleen was minced into small fragments and digested in 0.5 mg/mL Collagenase D, 4 μg/mL DNase I, and 2% FCS in RPMI (all from Sigma-Aldrich). Digested tissues were passed through a 70 μm cell strainer to generate single-cell suspensions. The splenocytes were incubated in Red Blood Cell Lysis Buffer Hybri-Max (Sigma-Aldrich) for 1 min at room temperature to lyse erythrocytes.

### Flow cytometry

The fluorochrome-conjugated antibodies used are listed in **Supplementary Table 1**. Where applicable, DN thymocytes were defined as B220^-^F4/80^-^TCRγδ^-^TCRβ^-^CD4^-^CD8α^-^CD11b^-^CD11c^-^NK1.1^-^Gr-1^-^. When analyzing non-thymocyte populations, cells were pre-incubated with anti-CD16/CD32 antibody to block Fc receptors. For CD34 staining, cells were incubated with anti-CD34 antibody at 37 °C for 15 min before staining with antibodies against all other cell-surface markers for 15 min on ice. For intracellular detection of transcription factors, cells were fixed and permeabilized with the Foxp3/Transcription Factor Staining Buffer Set (Thermo Fisher Scientific) when detecting transcription factors. Cell viability was analyzed using 4’,6-diamidino-2-phenylindole (DAPI, Sigma-Aldrich), propidium iodide, 7-Aminoactinomycin D (7-AAD, Thermo Fisher Scientific), or Live/Dead Fixable Yellow (Thermo Fisher Scientific).

The labeled cells were analyzed on a BD LSRFortessa II (BD Biosciences) or Cytek Aurora (Cytek Biosciences). Cell sorting was performed on either a BD FACSAria Fusion or BD FACSAria III Cell Sorter (BD Biosciences). Flow cytometry data were analyzed using FlowJo software (v10.10; Tree Star).

### CITE-seq sample preparation and sequencing

Anti-CD4 and anti-CD8α MACS-depleted thymocytes were labeled with TotalSeq-A anti-mouse sample hash tags (#155827, #155823 or #155825 from BioLegend) and stained with anti-c-KIT-PE-Cy7. Three hashtagged samples were generated: two from individual 12-week-old mice (one mouse per sample) and one from pooled thymocytes isolated from six 4–6-week-old mice. c-KIT^+^ cells were then FACS-purified from each sample and stained with the TotalSeq-A Mouse Universal Cocktail 1.0 (BioLegend, 199901) (**Supplementary Table 2**) diluted 1:5 in FACS Buffer (PBS, 0.5% FCS). 10,000 cells from each sample were then pooled, and these 30,000 cells were loaded across two 10x lanes on the Chromium controller using the Chromium Next GEM Single Cell 3’ HT Reagent Kit v3.1 (10x Genomics). Hashtag oligonucleotide (HTO), antibody-derived tag (ADT), and gene expression (GEX) sequencing libraries were prepared according to the manufacturer’s instructions. ADT libraries were sequenced on either the MGI T7 platform using the PE100 cycles Kit or the Illumina NovaSeq with 28 cycles for Read 1, 90 cycles for Read 2, and 10 cycles for the index read. GEX libraries were sequenced on the MGI T7 platform using the PE100 cycles Kit with 28 cycles for Read 1, 100 cycles for Read 2, and 10 cycles for the index read. The HTO library was sequenced on an Illumina NextSeq 2000 using a 100-cycle Kit: 87 cycles for Read 1, 30 for Read 2, and 10 for the index read.

### CITE-Seq and single-cell RNA-sequencing analysis

GEX, HTO, and ADT libraries were processed using the Cell Ranger multi-pipeline (v7.0.0), with GEX reads aligned to a combined mouse-human reference genome (refdata-gex-GRCh38_and_GRCm39-2024-A). All analyses were performed using Seurat (v5.4.0) (33). Sample identities were assigned using HTODemux, and only cells uniquely assigned to a single HTO were retained. Doublets were identified using Demuxafy (34) and removed from downstream analyses. Low-quality cells with ADT counts outside the median ± 5× median absolute deviation, mitochondrial content >5%, or with <400 or >5,500 detected genes were excluded.

RNA counts were log-normalized, while ADT and HTO counts were normalized across cells using the centered log-ratio (CLR) method with a margin of two. Principal component analysis was run on all genes using the standard Seurat pipeline. The ADT and RNA modalities were integrated using weighted nearest neighbor (WNN) analysis (35). The minimum number of principal components that explained >90% of the variation in gene expression in the data was used. The WNN graph was used to create the Uniform Manifold Approximation and Projection (UMAP).

Automated cell-type annotation was performed using the Bioconductor package SingleR (v2.12.0) (36), with the relevant populations from the Immunological Genome Project (ImmGen) mouse RNA-seq dataset (GSE109125) (19) as a reference. Cells were assigned to a cell cycle phase by calculating the cell cycle scores using the S and G2M phase-associated genes within the Seurat package.

Trajectory inference, including pseudotime analysis, was performed using the Bioconductor package Slingshot (37), with the gene expression matrix as input (38). The Python package scVelo’s dynamical model (39) was used for RNA velocity analysis.

A previously published 10X scRNA-seq dataset of ETP to DN2a thymocytes (GSE130812) (3) was also analyzed.

### Bulk RNA-seq sample preparation and analysis

Total RNA was extracted from sorted populations with the Arcturus PicoPure RNA Isolation Kit (Thermo Fisher Scientific). RNA quality checks were performed using a TapeStation (Agilent). Libraries were constructed using the TruSeq RNA Sample Prep Kit v2 (Illumina) and sequenced as paired-end (2 x 66 bp) on a NextSeq 2000 instrument (Illumina). The base calling and quality scoring were performed using Real-Time Analysis on-board software (v2.4.6), while the FASTQ file generation and de-multiplexing utilized bcl2fastq conversion software (v2.15.0.4).

After preprocessing, the reads were aligned to mm39 using STAR (v2.7.11b) (40) with default parameters. During alignment, gene-level read counts were determined using the --quantMode GeneCounts option and the Mus_musculus.GRCm39.110.gtf annotation file. All downstream analysis, including filtering and normalization, was performed using edgeR (v4.8.2) (41). Differentially expressed genes were identified using glmTreat with fold changes greater than or less than 2 and FDR < 0.05 as cutoffs (**Supplementary Table 3**). Subsequently, transcription factors were annotated using a curated list from the Aerts Lab cisTarget resource (https://resources.aertslab.org/cistarget/tf_lists/). RPKM values were calculated and log_2_-transformed prior to visualization using the R package pheatmap (v1.0.13). The relationships among samples were assessed using multidimensional scaling analysis with the plotMDS function from the edgeR package, with gene.selection = “common”.

### LoxCode analysis

Sorted substages of interest were lysed in DirectPCR lysis reagent (cell) (Viagen Biotech) with Proteinase K (ThermoFisher Scientific, 0.5mg/mL) and incubated for 2 h at 56 °C. The Proteinase K was then inactivated for 30 min at 85 °C and 5 min at 95 °C. LoxCode DNA was amplified using a 3-round PCR protocol (32). The libraries were pooled and sequenced using the Illumina NextSeq 2000 P1 Kit, 600 cycles (354 cycles for Read 1, 10 cycles for Index 1, 10 cycles for Index 2, 254 cycles for Read 2).

LoxCode elements derived from the raw Illumina NextSeq paired-end sequencing data were aligned to the original cassette using custom C++ code and the library edlib (42). Based on previously established methods, we determined the number of elements within each LoxCode (32) and the number of recombination steps required to generate it (complexity) (31). Except for the LoxCode alignment, all analyses were performed in R (version 4.5.2) and RStudio.

To retain only high-quality barcodes for downstream analyses, barcodes with complexity less than 3 (low complexity), repeated elements (possibly PCR artifacts), 13 elements (unrecombined), and barcodes with 10 or fewer reads across both PCR replicates were discarded. To enrich for barcodes less likely to be generated across two cells independently and more likely to be unique to each mouse, any barcodes found in 10 of the 37 independent samples from a previously generated empirical exclusion list (32) were also discarded. Each replicate was normalized to counts per million, and the counts for each sample were determined by averaging the PCR replicates. The average cosine similarity between samples was calculated from log(1+x)-transformed barcode counts using the R package coop (v0.6-3) and then normalized to the range 0 to 1. The values were then hierarchically clustered (method = “complete”) and visualized using pheatmap (v1.0.13). To visualize barcode distributions across samples, averaged counts per million values were column-normalized, and barcodes were ordered by their distribution patterns. The normalized values were then converted to a percentage of the maximum value within each sample, log(1+x)-transformed, and displayed using ComplexHeatmap (v2.26.1).

### OP9 co-culture assays

OP9-GFP and OP9-DL1 stromal cell lines were maintained in α-MEM medium (Thermo Fisher Scientific, 12561-056) supplemented with 20% FCS (Australian Origin, Bovogen Biological) and penicillin-streptomycin-glutamine (Thermo Fisher Scientific, 10378016). Before co-culture, the stromal cells were inactivated with 2.5 μg/mL mitomycin C for 1.5 h (Stem Cell). Sorted thymocyte subsets were then co-cultured on a monolayer of 6,000 stromal cells in 100 μl of medium in a 96-well plate for up to 7 (non-T assays) or 18 days (T-cell assays). For T-cell cultures maintained for up to 18 d, 1,000 cells were seeded per well. For analyses of DN substage progression at days 2 and 8, 10,000 cells were seeded per well. Myeloid cultures were initiated with 2,000 cells per well, whereas alternative-lineage and NK-cell cultures were initiated with 4,500 cells per well. T and alternative lineage cell cultures were supplemented with 5 ng/mL mouse IL-7 and 5 ng/mL human Flt3L. Myeloid cultures were supplemented with 1ng/mL mouse IL-7, 5ng/mL human Flt3L, 10ng/mL mouse SCF, 10ng/mL mouse G-CSF, 10ng/mL mouse GM-CSF, and 10ng/mL mouse IL-3. NK cell cultures were supplemented with 5ng/mL mouse IL-7, 10ng/mL human Flt3L, and 20ng/mL mouse IL-15. All cytokines were purchased from Peprotech. Non-T-cell cultures were also supplemented with 5 μM β-mercaptoethanol.

### Adoptive transfer of thymocytes

Thymocytes from six C57BL/6-CD45.1 mice were pooled, and 20,000 ETP or DN2a-1 thymocytes were FACS-purified and intravenously transferred (in 150 μL PBS) into one *Rag1*^−/−^ recipient mouse that was sublethally irradiated with 4-6 Gy. The spleen and thymus of individual recipient mice were then analyzed separately by flow cytometry 4 weeks post-transfer.

### Statistical analysis

Statistical analyses were performed in GraphPad Prism 10 (v10.4.1). Two-tailed unpaired Student’s *t*-test with Holm-Šídák’s multiple comparisons test, or one-way ANOVA with Tukey’s *post hoc* test for multiple comparisons, were performed.

## Results

### Single-cell multiomic profiling of c-KIT-expressing CD4^-^CD8^-^ DN thymocytes in the mouse thymus

To more precisely analyze the timing of T-lineage commitment, improved phenotypic resolution of DN2 thymocytes is needed. To achieve this, we employed CITE-seq, a single-cell multiomic approach that combines scRNA-seq with ADT profiling. In this study, we used a 128-antibody panel against cell-surface proteins (**Supplementary Table 2**). To capture cells along the ETP-to-DN2 developmental continuum, we FACS-purified c-KIT-expressing CD4^–^CD8^–^ cells from the thymus of C57BL/6 mice (**Supplementary Figure 1A**) for library construction. Across three replicate samples, we sequenced 7,758 cells, with an average of 2,651 genes/cell detected after quality control and removal of identified doublets (**Supplementary Figure 1B**). We first annotated the broad clusters using SingleR (36), based on transcriptional similarity to reference immune cell profiles from the ImmGen bulk RNA-seq database (19) (**Supplementary Figures 1C, D**). In addition to pre-T cells (DN thymocytes), we identified DCs, B cells, ILCs, and unconventional T cells. We removed these non-DN cells from downstream analysis.

We then re-clustered the *bona fide* 5,617 DN thymocytes and visualized them by UMAP, where cluster numbers reflected approximate developmental progression (**Figure 1A**). The 23 clusters included cells from all three replicates (**Supplementary Figure 2A**). To annotate these DN thymocytes, we first determined their similarity to the transcriptional profiles of DN stages in the ImmGen bulk RNA-seq database (19). Most cells were transcriptionally similar to ETP, DN2a, or DN2b thymocytes (**Figure 1B**). We then used the expression of selected markers previously used to define these stages (**Figures 1C, D**) to inform our final annotation (1) (**Figure 1E**).

**Figure 1:**
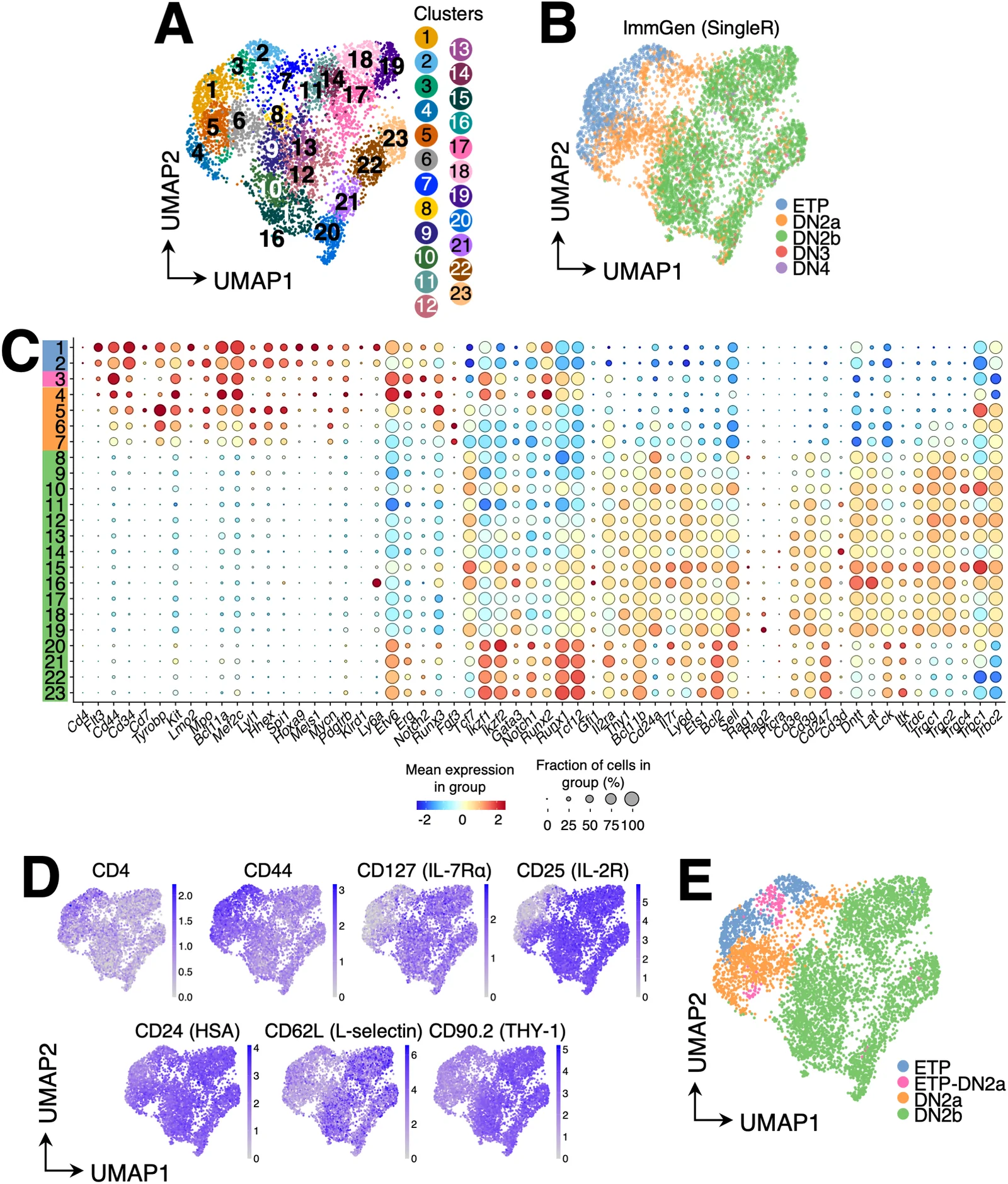
Single-cell multiomic map of early mouse thymocytes. **(A)** CITE-Seq analysis of c-KIT-expressing CD4^-^CD8^-^ DN thymocytes sorted from C57BL/6 mice (the gating strategy is shown in **Supplementary Figure 1A**). The data include cells from three independent biological samples. Shown is a WNN UMAP projection of 5,617 single cells, with 128 surface markers. The 23 clusters were identified using Seurat (resolution = 2). The numbering reflects approximate developmental progression. **(B)** Cells were annotated based on transcriptional similarity to the bulk RNA-seq datasets of DN thymocytes from ImmGen using SingleR. **(C)** Dot plot showing expression of selected non-T and T specification genes involved in T-cell development. Colored boxes around the cluster number indicate the assigned DN substage. **(D)** Expression of selected cell-surface markers that are canonically employed to assign the DN developmental stages. **(E)** Assignment of the clusters to DN substages based on the expression of selected stem and progenitor genes and T-lineage-associated genes.

Clusters 1–3 expressed high levels of *Cd44* RNA and CD44 protein as well as various HSPC-associated genes, including *Flt3*, *Cd34*, *Kit*, *Mef2c*, *Bcl11a*, and *Spi1* (1), but were CD25^lo^ (**Figures 1C, D**). Clusters 1 and 2 were characteristic of ETPs (**Figure 1E**) as they had some low-level CD4 expression (43), with CD24 expression lowest in cluster 1 (**Figures 1C, D**), a feature of the least mature ETPs (2). In contrast, *Il2ra* RNA expression was slightly increased in cluster 3 (**Figure 1C**), suggesting transition to the DN2a stage (1), and may include both ETP and DN2a thymocytes (**Figure 1E**).

Clusters 4–7 displayed characteristics of DN2a thymocytes (**Figure 1E**) with high CD44 expression but also higher *Il2ra* RNA and CD25 protein expression than clusters 1–3 (ETPs) (**Figures 1C, D**). Furthermore, expression of many HSPC-associated markers was retained, although at lower levels than clusters 1–3. Clusters 8–23 highly expressed *Il2ra* RNA and CD25 protein and had upregulated *Bcl11b* expression. However, expression of CD44 and HSPC-associated markers, including *Kit*, was downregulated compared to the other clusters (**Figures 1C, D**). They also highly expressed many other T-lineage-associated genes, including *Thy1* (encodes CD90), *Il7r*, *Ets1*, *Cd3e*, *Cd3g*, and *Cd247* (**Figure 1C**). Thus, clusters 8–23 displayed features of T-lineage committed DN2b thymocytes (**Figure 1E**). In particular, clusters 20–23 had high expression of *Tcf12* and Notch target genes, including those from the *Ikzf* and *Runx* family members, which are associated with the DN3a stage (1) (**Figure 1C**). However, clusters 20–23 lacked expression of other canonical DN3a genes, including *Ptcra*, *Rag1*, or *Cd3d* (**Figure 1C**). Thus, clusters 20–23 likely represent late-stage DN2b thymocytes (**Figure 1E**).

A final feature to note in our CITE-seq data was the definition of subsets based on cell-cycle phase (**Supplementary Figure 2B**). Cell proliferation is integral to T-cell development as it is required to generate enough thymocytes to establish a diverse T-cell repertoire (1). ETPs were mainly non-dividing (G1). However, cells in cluster 2 were predominantly defined by expression of proliferation-associated genes (e.g., *Mki67*, *Cdk1*, and *Birc5*) (**Supplementary Figure 2C**), consistent with prior studies showing that ETPs include both quiescent and proliferative subsets (3, 23). In contrast, both DN2a and DN2b cells were in various cell-cycle states (**Supplementary Figure 2B**), with cells in clusters 11, 14, 17–19, 22 and 23 highly expressing genes related to the S and G2/M phases of the cell cycle (**Supplementary Figure 2C**). This likely reflects responses to sequential signals that developing DN2 thymocytes receive during thymic development (1).

### Differences in *Thy1* (CD90) expression resolves pre- and post-T-lineage commitment DN2a thymocytes with higher resolution

Our CITE-seq data (**Figures 1A, E**) and prior studies indicate significant heterogeneity among ETPs, DN2a, and DN2b thymocytes. Notably, DN2a cells with distinct *Bcl11b* activation states were previously shown to differentially express numerous genes, including *Cd34* and *Thy1* (11). Consistent with these findings, our CITE-seq dataset revealed heterogeneous *Cd34* expression within DN2a thymocytes (**Figure 1C**). In addition, DN2a thymocytes appeared to express *Thy1* transcript and CD90 protein at a broader range than DN2b thymocytes, which uniformly expressed these at high levels (**Figures 1C, D**).

To confirm this heterogeneity, we next analyzed two replicates of a previously published scRNA-seq dataset of ETP to DN2 thymocytes from C57BL/6 mice, with DN3 cells included as a control (3) (**Supplementary Figures 3A, B**). Clusters were numbered to reflect approximate developmental progression (**Supplementary Figure 3A**), based on annotations from Zhou et al. (3), similarity of the transcriptional profile to the DN stages in the ImmGen bulk RNA-seq database (19) (**Supplementary Figure 3C**), and expression of key marker genes (**Supplementary Figure 3D**).

Clusters 9–13 corresponded to DN2a thymocytes, in which *Il2ra* was upregulated, but not *Ptcra*, *Cd3g*, *and Cd3d* (**Supplementary Figure 3D**) (1). The published dataset had more DN2a thymocytes, and we again observed heterogeneity among clusters annotated as DN2a (**Figures 1A, E**, **Supplementary Figure 3A**). As before, we could distinguish two subsets of DN2a thymocytes (**Supplementary Figure 3D**). The first subset (clusters 9 and 10) had low *Thy1* expression, but highly expressed *Cd34* and other HSPC-associated genes, including *Tyrobp*, *Bcl11a*, *Hhex*, and *Spi1*. Clusters 11–13, comprising the second subset, displayed high *Thy1* expression but lower expression of *Cd34* and other HSPC-associated genes. Importantly, clusters 11–13 showed increased *Bcl11b* expression (**Supplementary Figure 3D**).

DN2 thymocytes have been previously subdivided into c-KIT^hi^ DN2a and c-KIT^lo^ DN2b subsets, with Bcl11b-YFP upregulation coinciding closely with T-lineage commitment in DN2a thymocytes (9–11). Given the differences observed in *Cd34* and *Thy1* expression among DN2a thymocytes across both single-cell datasets (**Figure 1C**, **Supplementary Figure 3D**) (3), we next sought to resolve these two putative DN2a subsets by flow cytometry. Within total DN2 thymocytes, c-KIT expression displayed a reproducible bimodal distribution that was used to distinguish DN2a (c-KIT^hi^) and DN2b (c-KIT^lo^) cells. Among DN2a thymocytes, CD90 expression was similarly bimodal, enabling the discrimination of DN2a-1 (CD90^lo^) and DN2a-2 (CD90^hi^) subsets (**Figure 2A**, **Supplementary Figure 4**). Total DN2 thymocytes (CD44^+^CD25^+^) were therefore subdivided based on differential c-KIT and CD90 expression into DN2a-1 (c-KIT^hi^CD90^lo^), DN2a-2 (c-KIT^hi^CD90^hi^), and DN2b (c-KIT^lo^CD90^hi^) subsets (**Figure 2A**, **Supplementary Figure 4**). ETPs were defined as c-KIT^hi^ DN1 (CD44^+^CD25^-^) thymocytes (**Figure 2A**). Gating on these subsets, we could observe a decrease in CD34 expression as cells transitioned from the ETP to the DN2b stage, with DN2a-1 thymocytes expressing higher levels of CD34 than DN2a-2 thymocytes (**Figure 2B**). The changes in cell-surface protein expression (**Figures 2A, B**) were consistent with the changes in RNA expression we described earlier (**Figure 1C**, **Supplementary Figure 3D**). We thus concluded that these markers are useful for resolving two DN2a subsets by flow cytometry (**Figure 2C**).

**Figure 2:**
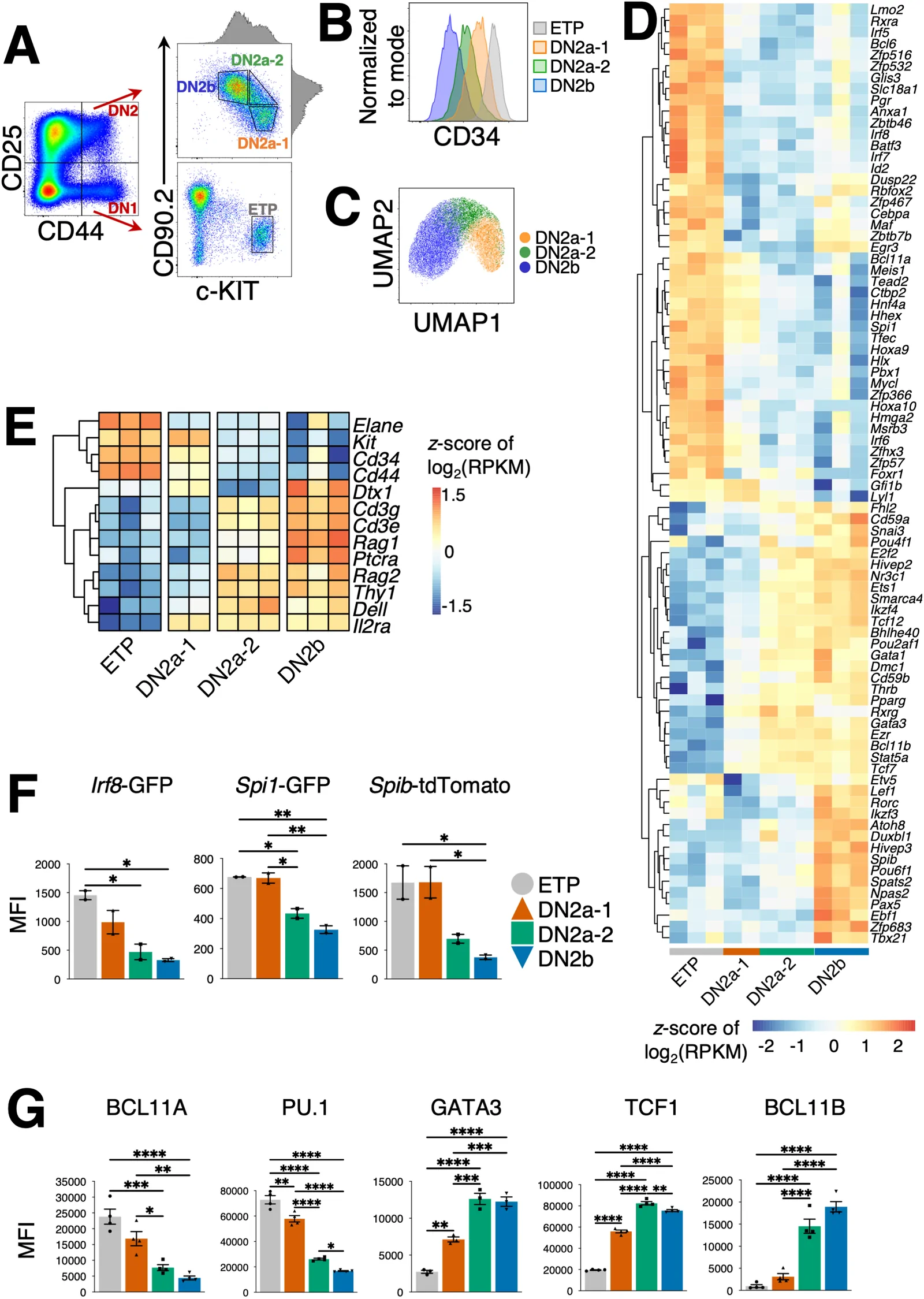
Differential expression of c-KIT and CD90 resolves an additional DN2 subset. **(A)** Representative gating strategy for identifying ETPs (within the CD44^+^CD25^-^ DN1 gate) and subdividing CD44^+^CD25^+^ DN2 thymocytes in the thymus of C57BL/6 mice. **(B)** Comparison of CD34 expression by ETPs and DN2 subsets. **(C)** UMAP visualization of the flow cytometric analysis of DN2 thymocytes for CD44, CD25, c-KIT, CD90, and CD34 expression. **(D)** Bulk RNA-seq analysis of ETP and DN2 subsets. Shown is the heatmap of differentially expressed transcription factor genes (FDR < 0.05, minimum 2-fold change) identified using glmTreat in edgeR. **(E)** Heatmap of the same bulk RNA-seq data for selected genes associated with T-cell development. **(F)** Flow cytometry analysis of ETPs and the DN2 subsets for *Irf8*-GFP, *Spi1*-GFP, or *Spib*-tdTomato expression in reporter mice, with each dot representing an individual mouse. **(G)** Flow cytometry analysis of ETPs and the DN2 subsets for BCL11A, PU.1, GATA3, TCF1, and BCL11B protein expression by intracellular staining, with each dot representing an individual mouse. Shown are the mean ± SEM of the mean fluorescence intensity (MFI) for each reporter or protein. One-way ANOVA with Tukey’s *post hoc* test for multiple comparisons. *P < 0.05, **P < 0.01, ***P < 0.001, ****P < 0.0001.

### Transcriptional dynamics distinguish DN2 subsets through differential expression of multipotency and T-lineage-associated markers

With this new DN2a subset gating strategy (**Figure 2A**), we next performed bulk RNA-seq on sorted ETP, DN2a-1, DN2a-2, and DN2b thymocytes to obtain more in-depth transcriptional information. Bulk RNA-seq was used to complement the single-cell analyses, as low-abundance transcripts, including many transcription factors, are susceptible to dropout in scRNA-seq datasets (44).

At the transcriptional level, DN2a-1 thymocytes aligned more closely with ETPs, while DN2a-2 thymocytes were more similar to DN2b thymocytes (**Figure 2D**). Congruent with gene expression patterns observed at the single-cell level (**Figure 1C**, **Supplementary Figure 3D**), ETPs and DN2a-1 thymocytes expressed higher levels of HSPC-associated genes, including *Cd34, Cd44*, and *Kit*, but had lower expression of T-lineage genes, including *Thy1*, in comparison to DN2a-2 and DN2b thymocytes (**Figures 2D, E**). Importantly, *Il2ra* was expressed in the DN2 subsets (**Figure 2E**), indicating that the DN2a-1 subset is unlikely to be ETPs.

Networks of transcription factors are central to T-cell developmental progression, particularly in establishing T-cell identity and suppressing alternative lineage fates (1). We therefore examined the expression dynamics of key regulators across the ETP-to-DN2 continuum. *IRF8* has been associated with DC-primed progenitors in the human thymus, but the same study found that IRF8 protein was not detected in mouse thymic progenitors (45). Nevertheless, *Irf8* expression was highest in ETPs and was downregulated upon transition to the DN2a-1 stage (**Figure 2D**). Two additional multipotency-associated transcription factors, *Bcl11a* and *Spi1*, were highly expressed in ETPs and progressively downregulated across the ETP-to-DN2b continuum, with the most pronounced decrease occurring at the DN2a-2 and DN2b stages. Consistent with its role in T-lineage commitment (11), *Bcl11b* expression increased across development, from low levels in ETPs to maximal expression in DN2a-2 and DN2b thymocytes. *Tcf7* and *Gata3*, which are indispensable for T-lineage identity (1), displayed broadly similar expression patterns, although *Tcf7* was already highly expressed in DN2a-1 thymocytes. Lastly, *Spib*, which is transiently induced following T-lineage commitment (46), was upregulated specifically at the DN2b stage (**Figure 2D**).

To validate the RNA expression of these transcription factors, we first employed reporter mice (**Figure 2F**, **Supplementary Figure 5A**). Consistent with the bulk RNA-seq data (**Figure 2D**), *Irf8*-GFP expression was highest in ETPs, followed by DN2a-1 thymocytes and downregulated in DN2a-2 and DN2b thymocytes (**Figure 2F**). Similar to the *Spi1* (encodes PU.1) RNA expression (**Figure 2D**), *Spi1*-GFP expression was high in both ETP and DN2a-1 subsets, before decreasing in DN2a-2 and DN2b thymocytes (**Figure 2F**). In contrast to *Spib* RNA expression, which increased at the DN2b stage (**Figure 2D**), *Spib*-tdTomato expression was higher in ETP and DN2a-1 thymocytes than in DN2a-2 and DN2b thymocytes (**Figure 2F**). This discordance indicates that reporter activity does not fully recapitulate endogenous *Spib* expression across early thymocyte development, potentially owing to differences in transcriptional and protein kinetics or to the persistence of reporter expression following earlier activation of the *Spib* locus.

Because gene expression does not always correlate with protein expression, we performed flow cytometric analysis to assess expression of key regulators (**Figure 2G**, **Supplementary Figures 5B-E**). BCL11A and PU.1 protein (encoded by *Spi1*) were highly expressed in ETPs and DN2a-1 thymocytes but significantly reduced in DN2a-2 and DN2b thymocytes (**Figure 2G**, **Supplementary Figures 5B, C**). Expression of T-lineage-associated factors GATA3 and TCF1 (encoded by *Tcf7*) was significantly higher in DN2a-1 thymocytes than in ETPs (**Figure 2G**, **Supplementary Figures 5C, D**). Notably, a subset of DN2a-1 thymocytes co-expressed BCL11A and BCL11B (**Supplementary Figure 5B**), indicating initiation of the T-lineage transcriptional program and a transitional developmental state between multipotency and T-lineage commitment (1). In contrast, GATA3, TCF1, and BCL11B were highly expressed in DN2a-2 and DN2b thymocytes (**Figure 2G**, **Supplementary Figures 5B-D**).

Overall, ETPs highly expressed multipotency-associated markers at both the RNA and protein levels, including *Irf8*, *Spi1* (PU.1), and *Bcl11a* (**Figures 1C**, **2D–G**, **Supplementary Figure 3D**). Relative to ETPs, these factors were expressed at slightly lower levels in DN2a-1 thymocytes. Concurrently, DN2a-1 thymocytes upregulated markers associated with the T-lineage transcriptional program, most notably *Tcf7* (TCF1). In contrast, the DN2a-2 subset displayed diminished expression of HSPC-associated markers, accompanied by a marked upregulation of T-lineage-associated markers, including *Bcl11b*, *Gata3* and *Tcf7*, whose expression increased further in DN2b thymocytes. Thus, DN2a-1 thymocytes are characterized by co-expression of multipotency and T-lineage-associated factors. In contrast, DN2a-2 thymocytes showed reduced expression of multipotency-associated factors along with increased expression of T-lineage-associated genes, indicative of a predominantly T-lineage-committed transcriptional profile (**Figures 1C**, **2D–G**, **Supplementary Figure 3D**) (1).

### *In vivo* clonal dynamics reveal a shared developmental pathway

To investigate the developmental relationships between ETPs and the DN2 subsets, we performed trajectory inference analysis on our CITE-seq data using the Slingshot algorithm (37) (**Figure 3A**). This predicted several trajectories originating from cluster 1. However, the longest trajectory suggested a developmental progression from ETP to DN2a to DN2b (**Figure 3A**), consistent with the observed increase in pseudotime values along this pathway (**Figure 3B**). Similar developmental kinetics were also predicted using RNA velocity (39) (**Supplementary Figure 6A**). Similarly, a trajectory inferred from the bulk RNA-seq data suggested progression from ETP to DN2a-1, then to DN2a-2, and finally to the DN2b thymocytes (**Figure 3C**).

**Figure 3:**
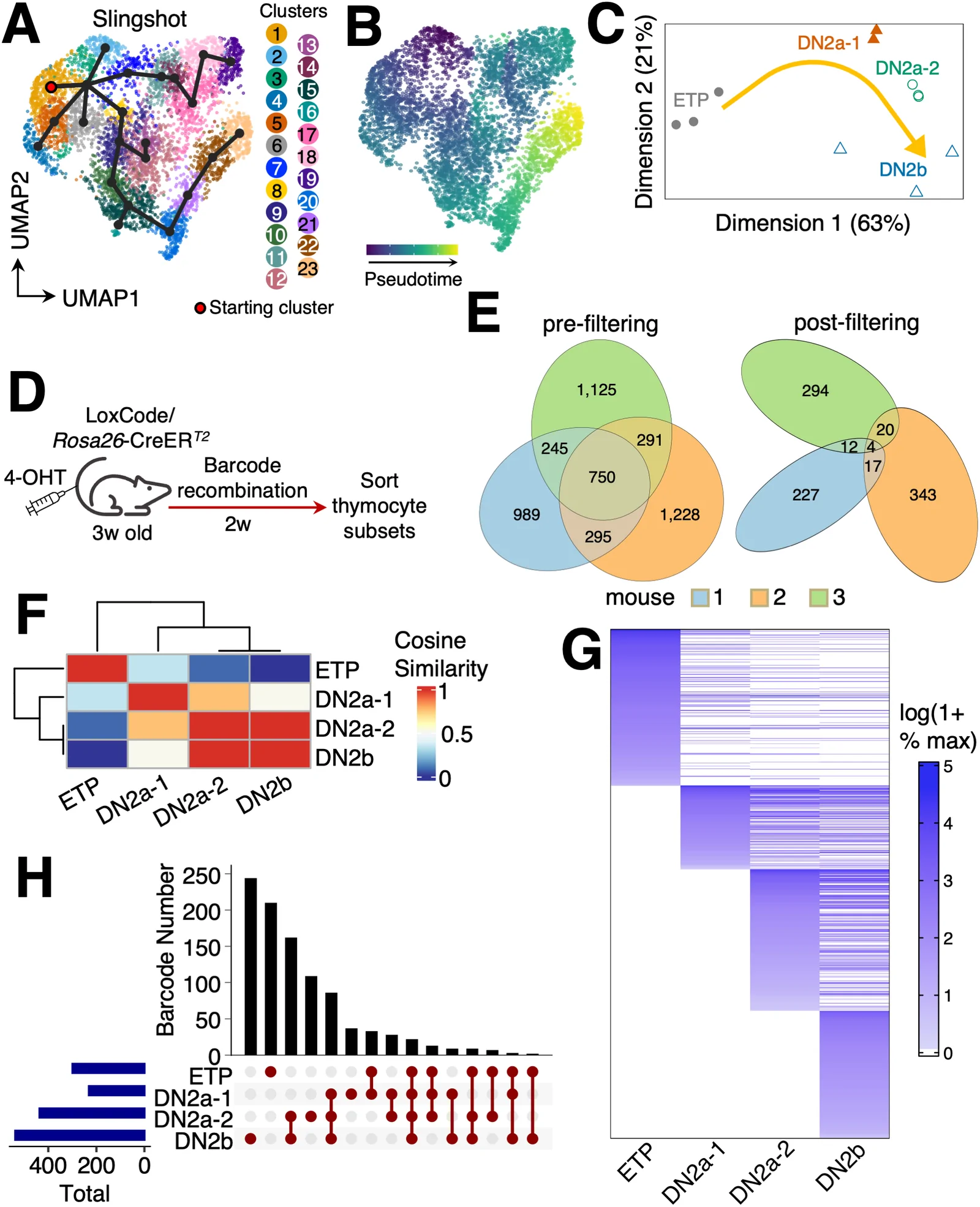
ETP, DN2a-1, DN2a-2, and DN2b thymocytes are on a shared developmental continuum. **(A)** Developmental trajectories inferred by Slingshot of the CITE-seq dataset of early thymocytes, with cells color-coded by Seurat clusters. **(B)** Pseudo-temporal ordering of cells along the inferred Slingshot trajectory. **(C)** Multidimensional scaling analysis of bulk RNA-seq datasets of ETP and DN2 subsets. Shown is the projection of biological replicates along the first two dimensions based on commonly expressed genes. The arrow indicates the T-cell developmental trajectory. **(D)** LoxCode/*Rosa26^Cre-ERT2^* mice were treated with 4-OHT to induce barcode recombination. After 2 weeks, ETP, DN2a-1, DN2a-2, and DN2b thymocytes from three individual mice were FACS-purified separately for barcode analysis. **(E)** The number of detected barcodes per mouse before and after filtering for high-quality barcodes, including the extent of overlap between mice. **(F)** Hierarchically clustered heatmap of cosine similarity among detected barcodes across the indicated DN substages in the thymus. **(G)** Distribution of barcodes across ETP, DN2a-1, DN2a-2, and DN2b subsets. Barcodes are ordered by rank and colored by the relative abundance in each subset. **(H)** UpSet plot of barcode sharing between the indicated substages, with the total number of barcodes in each population shown.

To further investigate the developmental dynamics of ETPs and the DN2 subsets, we performed *in vivo* lineage tracing using the progeny of Cre-LoxP-based LoxCode mice (32), crossed to *Rosa26*-CreER^T2^ mice (**Figure 3D**). In this model, Cre induction triggers LoxCode recombination, generating highly diverse, heritable barcodes that uniquely label individual cells and their progeny (32). ETPs, DN2a-1, DN2a-2, and DN2b thymocytes were isolated from the thymus of these mice 2 weeks after 4-OHT administration for barcode analysis (**Figure 3D**). Barcode recovery was consistent across the three mice, yielding 4,923 barcodes in total, of which 974 passed quality filtering (**Figure 3E**). Of the filtered barcodes, no more than 2% were shared between two mice, and only four barcodes were shared across all three biological replicates, indicating that the vast majority were *bona fide* single-use barcodes (**Figure 3E**). We found the highest cosine similarity values, an indicator of relatedness, between DN2a-2 and DN2b thymocytes, though DN2a-1 thymocytes were also more similar to their DN2 counterparts than to ETPs (**Figure 3F**). The results were reproducible across all three mice (**Supplementary Figure 6B**).

Hierarchical clustering of the detected barcodes similarly revealed the proximity of the DN2 subsets, particularly DN2a-2 and DN2b thymocytes (**Figure 3G**). Most barcodes (62%) were only present in one subset (**Figures 3G, H**). Adoptive transfer of bone marrow-derived progenitors and computational modeling have previously shown that ETPs take approximately 9 to 12 days to progress to the subsequent DN2a stage (47, 48). Thus, within the 2 weeks, only the more mature ETPs were likely to have progressed to the DN2a and DN2b stages, which might explain the high number of unique barcodes within ETPs. Furthermore, unique barcodes may reflect cells that have fully transitioned to subsequent stages, such that these barcodes could not be detected in the precursor population. Despite this, we observed extensive barcode sharing among at least two of the DN subsets analyzed, with a few barcodes shared across all four subsets (**Figures 3G, H**). Altogether, these results suggest that, *in vivo*, ETP, DN2a-1, DN2a-2 and DN2b thymocytes are along a shared developmental continuum.

### T-cell developmental kinetics of ETPs and DN2 substages *in vitro*

To experimentally demonstrate the developmental relationship between ETPs and the three DN2 subsets, we investigated their *in vitro* differentiation kinetics using the OP9-DL1 co-culture system. αβ T-lineage developmental kinetics were tracked by measuring CD8 expression, which is upregulated during the CD8^+^TCRβ^lo^ immature single-positive (ISP) and double-positive (DP) stages (**Figure 4A**, **Supplementary Figure 7A**). By days 6 and 10 of culture, most DN2b cells had differentiated into CD8^+^ thymocytes, whereas only a smaller fraction of DN2a-2 cells reached this stage (**Figure 4A**, **Supplementary Figure 7B**). In contrast, progeny derived from ETP and DN2a-1 thymocytes remained CD8^-^. By days 14 and 18 of culture, a subset of ETP- and DN2a-1-derived progeny had become CD8^+^. However, these cultures lagged behind DN2a-2 and DN2b cultures, which contained significantly higher proportions of CD8^+^ thymocytes (**Figure 4A**, **Supplementary Figure 7B**).

**Figure 4:**
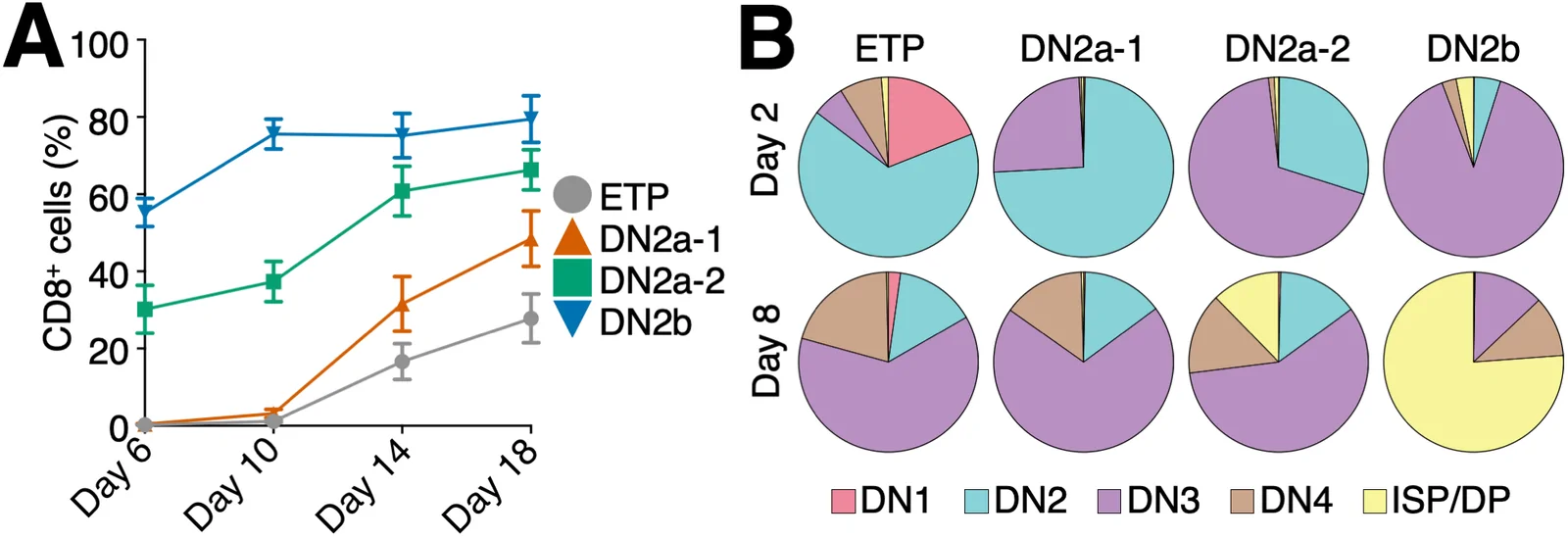
The αβ developmental kinetics of ETPs and DN2 subsets *in vitro* suggest a sequential developmental pathway. We sorted ETPs or DN2 subsets from the thymus of C57BL/6 mice and co-cultured them on OP9-DL1 stromal cells. We analyzed the progeny by flow cytometry at the indicated time points. **(A)** αβ T-lineage differentiation was assessed by quantifying CD8^+^ thymocytes, which identify CD8^+^TCRβ^lo^ ISP and DP cells (the gating strategy is shown in **Supplementary Figure 6A**). The mean ± SEM (n = 14 individual wells from 7 independent experimental repeats) is shown. **(B)** Analysis of DN substages and CD8^+^TCRβ^lo^ ISP + DP thymocytes, with pie charts showing the fraction of cells in the culture at each thymocyte substage (the gating strategy is shown in **Supplementary Figure 8A**). The data shown are the mean of five individual wells from two independent experimental repeats.

We next assessed progression through the DN stages at days 2 and 8 of culture (**Figure 4B**, **Supplementary Figures 8A–C**). By day 2, more than half of ETPs and DN2a-1 cells remained at the DN1 or DN2 stage, whereas most DN2a-2 and DN2b cells had progressed to the DN4 stage (**Figure 4B**, **Supplementary Figure 8C**). DN4-stage cells were not detected in ETP or DN2a-1 cultures until day 8 (**Figure 4B**, **Supplementary Figure 8C**). Consistent with previous reports that ETPs can require up to 12 days to transition to the next stage (48), we found that a proportion of ETP-derived thymocytes remained at the DN1 stage even by day 8 *in vitro* (**Figure 4B**). Overall, the *in vitro* differentiation kinetics aligned closely with both the predicted developmental trajectories (**Figure 3A**, **Supplementary Figure 6A**) and the clonal relationships revealed by the LoxCode model (**Figures 3F-H**), supporting the conclusion that ETPs, DN2a-1, DN2a-2 and DN2b thymocytes constitute a developmental continuum. Moreover, the progressive acceleration in T-lineage developmental kinetics across these substages is consistent with a linear developmental progression from ETPs through DN2a-1 and DN2a-2 to DN2b thymocytes.

### CD90 upregulation aligns with reduced multilineage potential in DN2a thymocytes

The prevailing model proposes that DN2a thymocytes retain multilineage potential, whereas DN2b thymocytes are committed to the T-lineage (2, 11). We therefore investigated whether the transition from the DN2a-1 to DN2a-2 substage might coincide more closely with the commitment checkpoint. To address this, we first assessed the non-T-lineage potential of the ETP and DN2 substages in culture conditions lacking Notch ligand (**Figure 5A**, **Supplementary Figures 9A, B**), and then with the addition of cytokines that drive NK- (**Figure 5B**, **Supplementary Figures 9C, D**) or myeloid-lineage differentiation (**Figure 5C**, **Supplementary Figures 9E, F**). In line with previous findings (4–7), only ETPs gave rise to B220^+^/CD19^+^ B-lineage cells, while both ETPs and DN2a-1 thymocytes differentiated into NK1.1^+^/DX5^+^ NK-lineage cells (**Figures 5A,B**). In contrast, DN2a-2 and DN2b thymocytes lacked B- and NK-lineage potential (**Figures 5A, B**).

**Figure 5:**
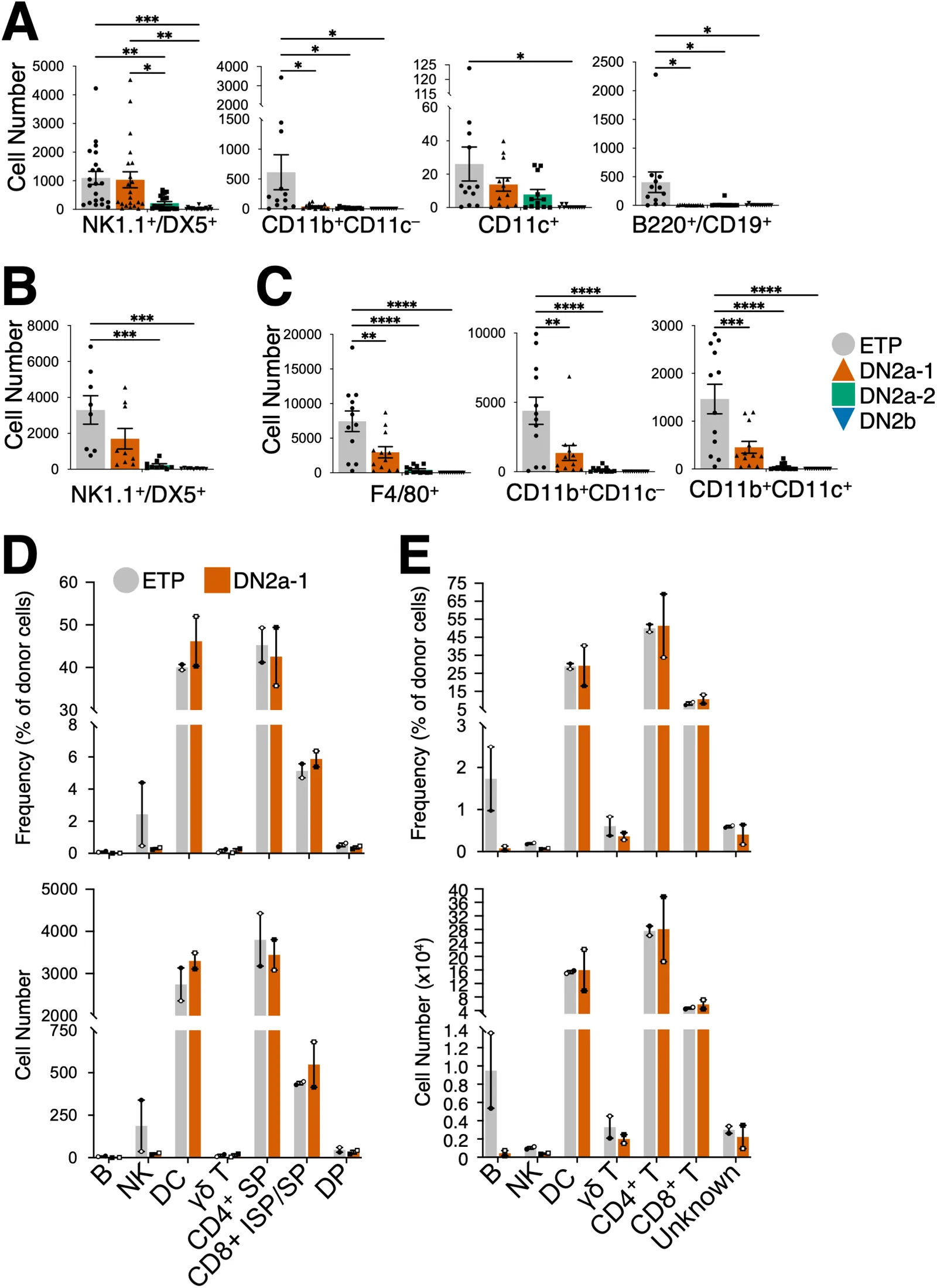
The ability of thymocytes to give rise to non-T cells is reduced during the DN2a-1 to DN2a-2 transition. ETPs or DN2 substages were sorted from the thymus of C57BL/6 mice and co-cultured on OP9 (without DL1) stromal cells for 7 days, and differentiation was analyzed by flow cytometry. NK1.1 and/or DX5 expression identified NK cells, CD11b identified total myeloid cells, CD11c identified DCs, B220 and/or CD19 (and lack of other lineage markers) identified B cells, while F4/80 identified macrophages (the gating strategies are shown in **Supplementary Figures 9A, C, E**). Shown are the number of cells detected in cultures that support the differentiation of **(A)** non-T (IL-7, Flt3L), **(B)** NK (IL-7, Flt3L, IL-15), and **(C)** myeloid (IL-7, Flt3L, SCF, G-CSF, GM-CSF, IL-3) lineage cells. The mean ± SEM are shown, with each dot representing an individual well from at least three independent experimental repeats for **(A, B)** and four independent experimental repeats for **(C)**. One-way ANOVA with Tukey’s *post hoc* test for multiple comparisons. *P < 0.05, **P < 0.01, ***P < 0.001, ****P < 0.0001. ETPs or DN2a-1 thymocytes from CD45.1 mice were adoptively transferred into sublethally irradiated *Rag1*^-/-^ (CD45.2^+^) recipient mice, then the **(D)** thymus and **(E)** spleen were analyzed for immune cells derived from the transferred cells (gating strategy shown in **Supplementary Figure 10**). Shown are the frequency and cell numbers of the indicated lineages within the CD45.1^+^ population from each tissue. Mean ± SEM of two independent experiments are shown. Each dot represents an individual recipient mouse. Circles and squares indicate the donor thymocyte subset (ETP or DN2a-1), while open and closed symbols indicate the independent experimental repeats. Two-tailed unpaired Student’s *t*-test with Holm-Šídák’s multiple comparisons test. No statistical difference found.

ETPs exhibited the greatest myeloid potential, which was progressively diminished in DN2a-1 and DN2a-2 cells before being completely lost at the DN2b stage (**Figures 5A, C**). Although DN2a-2 cells gave rise to a detectable number of CD11c^+^ cells in the absence of Notch signaling (**Figure 5A**), they failed to differentiate into myeloid-lineage cells under myeloid-promoting conditions (**Figure 5C**), suggesting that by the DN2a-2 stage, thymocytes had largely lost myeloid potential.

Thus, consistent with prior studies (3–6, 8, 23), ETPs are multipotent, whereas DN2a-1 thymocytes retain NK- and myeloid-lineage potential but not B-lineage potential. In contrast, DN2a-2 thymocytes have predominantly lost non-T-lineage potential and display markedly reduced myeloid potential, although they retain the capacity to differentiate into CD11c^+^ cells under some conditions. Non-T-lineage potential is completely absent in DN2b thymocytes. Together, these findings indicate that CD90 upregulation during the DN2a-1 to DN2a-2 transition closely coincides with T-lineage commitment.

To further support this finding that ETPs and DN2a-1 thymocytes are multipotent, we sorted these two populations from CD45.1^+^ mice and adoptively transferred them into sublethally irradiated CD45.2 *Rag1*^−/−^ mice. We analyzed the phenotype of cells derived from the CD45.1^+^ donors 4 weeks later (**Figures 5D, E**, **Supplementary Figures 10A, B**). Transferred ETPs and DN2a-1 thymocytes both gave rise to αβ T cells, γδ T cells, CD11c^+^ DCs, and NK cells in the thymus and spleen, although NK-cell frequencies were lower following transfer of DN2a-1 cells. Consistent with the *in vitro* OP9 coculture experiments, transfer of ETPs, but not DN2a-1 thymocytes, resulted in B cells, which were observed in the spleen. Thus, the lineage outcomes in this adoptive-transfer system align with the results of the *in vitro* differentiation assays. Together, these data suggest that the DN2a-1 to DN2a-2 transition coincides with the commitment checkpoint.

### PU.1, but not SPI-B, is required for early T-cell development

Given the dynamic expression patterns of *Spi1* and *Spib* during progression from the ETP to DN2b stage (**Figures 1C**, **2D, F, G**), we investigated the roles of PU.1 and SPI-B during T-cell development. Germline deficiency or hematopoietic-specific knockout of PU.1 affects HSPCs as well as immature thymocytes (49), making it difficult to distinguish prethymic from intrathymic effects (25, 50). To limit the impact of PU.1 deficiency on upstream progenitors, we analyzed the thymus of *Spi1^fl/fl^Rosa26*-CreER^T2^ conditional knockout mice one week after the initiation of 4-OHT treatment (**Figures 6A, B**) as this interval is sufficient for mature ETPs to progress to the DN2 or DN3 stage (47, 48).

**Figure 6:**
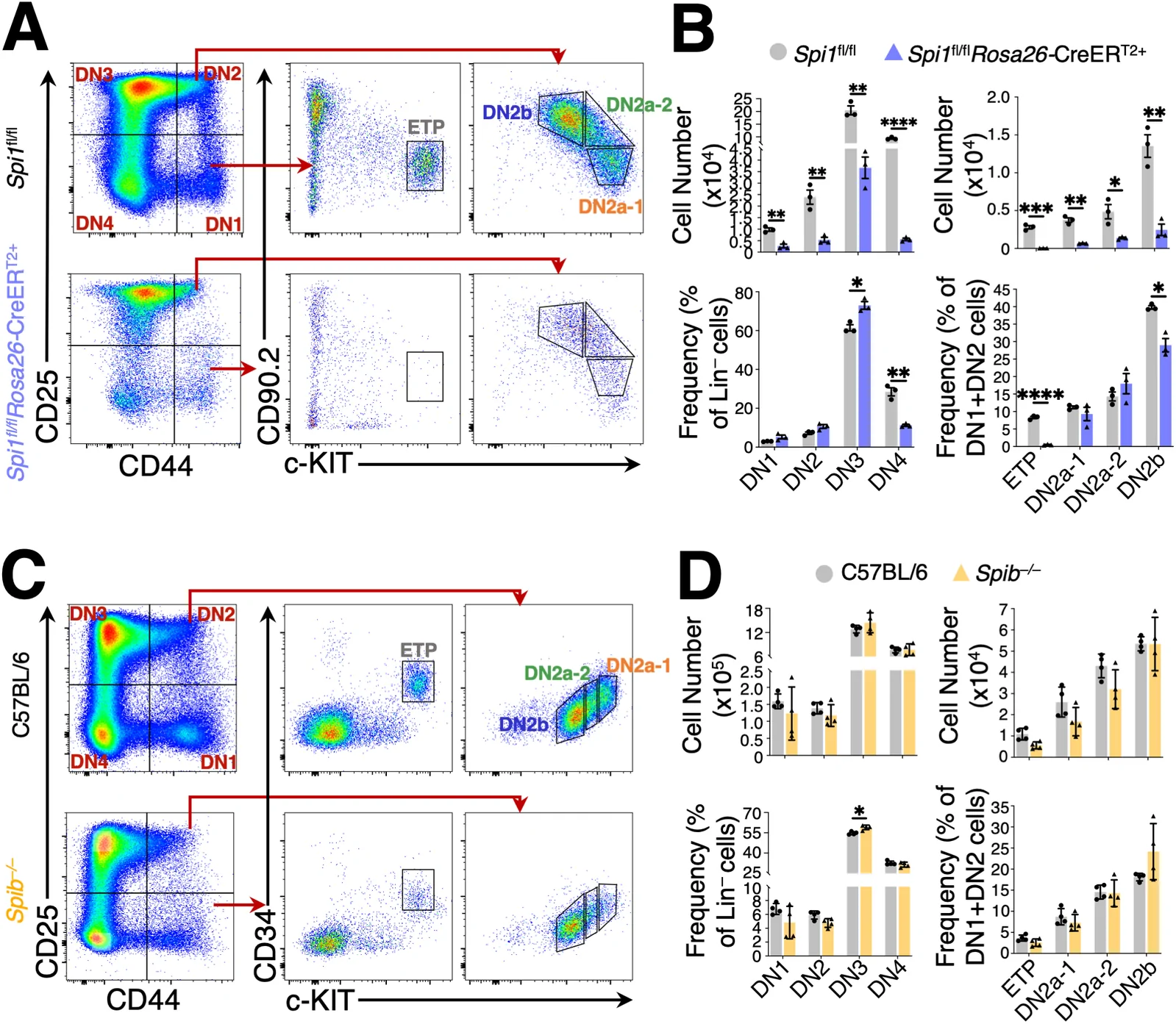
PU.1 deficiency, but not SPI-B deficiency, impairs early T-cell development. **(A)** *Spi1^fl/fl^* and *Spi1^fl/fl^Rosa26*-CreER^T2+^ mice were injected with 4-OHT at 17 and 21 days of age, then the thymi were harvested at 24 days of age for analysis by flow cytometry. Shown is a representative analysis of the DN thymocytes. **(B)** Analysis of individual animals for the number (top) and frequency (bottom) of each substage. The mean ± SD is shown, where each dot represents an individual mouse. **(C)** The thymus of *Spib*^-/-^ and C57BL/6 wild-type mice was analyzed by flow cytometry. Shown is a representative analysis of the DN thymocytes. **(D)** Analysis of individual animals for the number (top) and frequency (bottom) of each substage analyzed. The mean ± SD are shown, with each dot representing an individual mouse. Two-tailed unpaired Student’s *t*-test with Holm-Šídák’s multiple comparisons test. *P < 0.05, **P < 0.01, ***P < 0.001, ****P < 0.0001.

Consistent with previous studies showing impaired proliferation of PU.1-deficient thymic precursors *in vitro* (13, 14), we observed a significant decrease in the absolute number of all DN thymocyte substages, with ETPs showing the most pronounced reduction (**Figure 6B**). In contrast, the frequency of DN3 thymocytes increased, whereas DN4 thymocytes decreased (**Figure 6B**). These findings are consistent with accelerated progression from the DN2 to DN3 stage following loss of PU.1 (13, 14). PU.1-deficient mice also exhibited reduced CD34 expression among ETP and DN2 thymocytes (**Supplementary Figure 11A**).

The role of SPI-B in specific stages of T cell development remains less well defined. As CD90 expression differed between wild-type and *Spib^-/-^* DN thymocytes (**Supplementary Figure 11B**), we identified DN2 substages based on CD34 and c-KIT expression (**Figure 6C**). Using this alternate gating strategy, we detected no differences in ETP, DN2, or later developmental stages between wild-type and SPI-B-deficient mice (**Figure 6D**, **Supplementary Figures 11C, D**). These findings are consistent with previous reports showing that SPI-B deficiency in mice does not impact overall thymic cellularity (26).

Together, these results suggest that PU.1 is required for DN thymocyte proliferation and normal developmental progression (**Figure 6B**). By contrast, despite altered CD90 expression in SPI-B-deficient mice among DN thymocytes, loss of SPI-B had no discernible effect on T-cell development (**Figure 6D**, **Supplementary Figure 11D**).

## Discussion

We confirmed the previously reported heterogeneity among DN2a thymocytes (3, 11, 23) and established two subpopulations, DN2a-1 and DN2a-2, identified by cell-surface marker phenotype. DN2a-1 thymocytes retained multilineage potential, whereas DN2a-2 thymocytes were largely committed to the T-lineage. These findings identify DN2a-1 and DN2a-2 as intermediates along the T-cell developmental pathway. Transcriptional profiling and T-cell developmental kinetics revealed that DN2a-1 thymocytes are more closely related to ETPs, whereas DN2a-2 thymocytes are more related to DN2b thymocytes. This distinction has important implications because DN2a thymocytes have traditionally been treated as a single developmental population. Together, our data show that commitment to the T-cell lineage closely coincides with the DN2a-1 to DN2a-2 transition, identifying a previously unrecognized intermediate stage in early T-cell development.

Previous studies associated activation of *Bcl11b* in DN2a thymocytes with T-lineage commitment (11); however, surface markers capable of resolving these developmental states were lacking. CD90 expression has previously been reported to progressively increase from the ETP to DN4 stage (12), and *Cd34* is highly expressed in ETPs (3, 11). Notably, we found that these markers effectively resolve DN2a thymocytes into c-KIT^hi^CD90^lo^CD34^hi^ DN2a-1 and c-KIT^hi^CD90^hi^CD34^int^ DN2a-2 thymocytes (**Figure 7**). Both ETPs and DN2a-1 cells co-expressed HSPC- and T-lineage-associated markers, consistent with multilineage priming (3), whereas DN2a-2 and DN2b thymocytes expressed gene and protein signatures that are predominantly characteristic of T-lineage-committed thymocytes (1).

**Figure 7:**
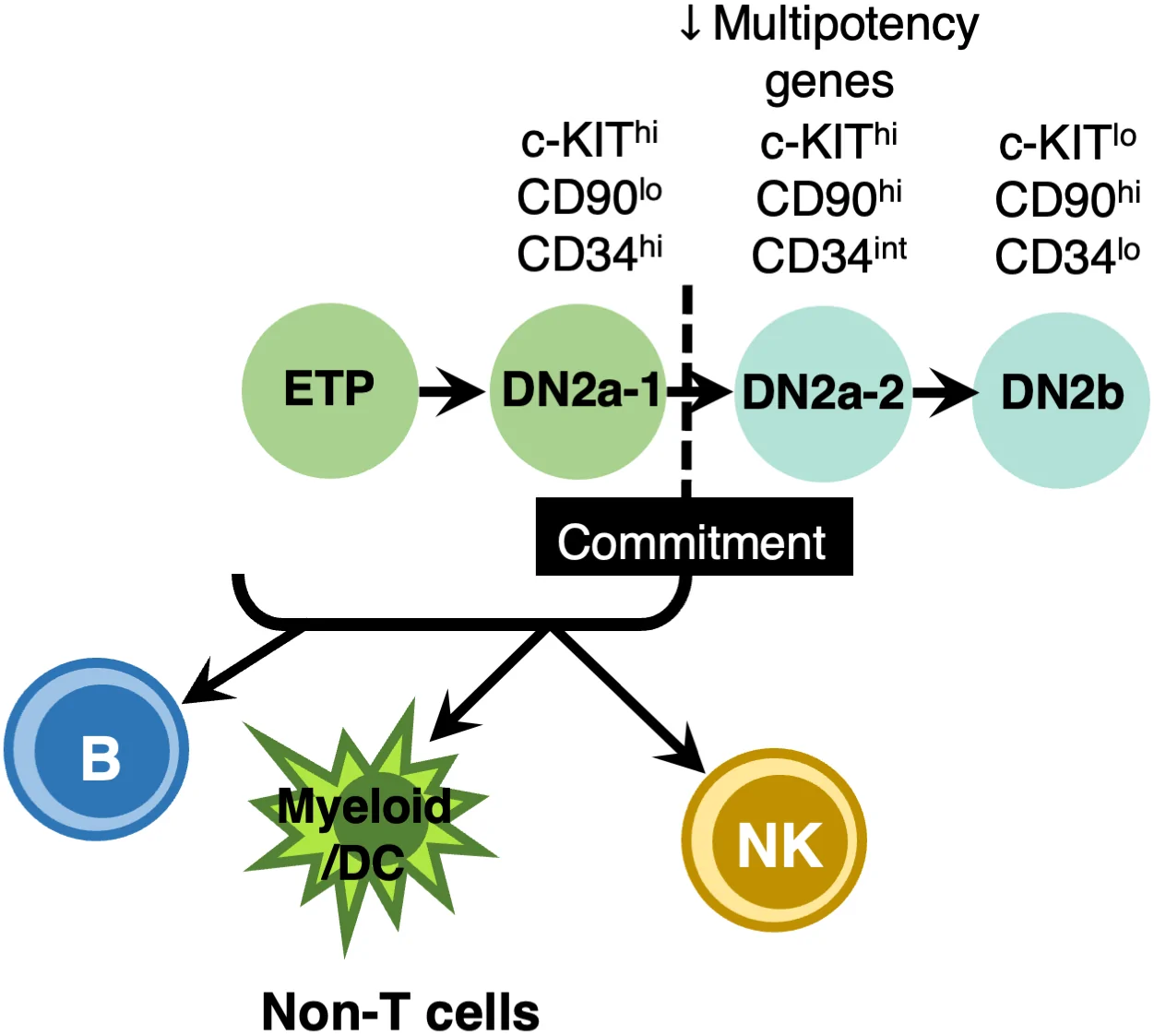
Differential expression of c-KIT, CD90, and CD34 resolves pre- and post-T-lineage-committed DN2 cells. The transition from the DN2a-1 to DN2a-2 substage coincides with the upregulation of CD90, downregulation of multipotency-associated factors, and loss of alternative lineage potential. This closely coincides with thymocyte commitment to the T-lineage.

Although *in vitro* systems, such as OP9-DL1 co-cultures (51), have been invaluable for understanding T-cell development, they cannot fully recapitulate the complexity of the thymic microenvironment. Thus, data from our LoxCode mouse model are important for inferring clonal relationships *in vivo*, indicating that ETPs and DN2 substages lie along a shared developmental continuum. The presence of numerous unique barcodes within each substage likely reflects the continuous and asynchronous nature of early T-cell development, whereby individual thymocytes progress through successive developmental states at variable rates (48).

Our findings, together with previous studies, show that thymocytes co-expressing T and alternative genes can differentiate into non-T lineages *in vitro* in the absence of Notch signaling (3, 5–7, 10, 23). Whether ETPs retain B-lineage potential has been controversial (4, 8), with our data suggesting that ETPs retain B-lineage potential, which is subsequently lost at the DN2a-1 stage. In addition, although granulocyte- or myeloid-committed precursors have been identified in the thymus (3, 23), their developmental relationship to ETPs remains unclear (23). We show that ETPs and DN2a-1 thymocytes retain both NK and myeloid lineage potential, supporting their classification as multipotent thymocytes. However, we also found that alternative lineage potential is not completely diminished by the DN2a-2 stage, as they can still give rise to CD11c^+^ myeloid cells under certain *in vitro* culture conditions. These observations also raise the question of whether alternative lineages develop in the thymus under physiological conditions or instead migrate into the thymus as differentiated populations. This is particularly important because increasing evidence suggests that thymic progenitors may undergo T-lineage priming before entering the thymus (52). Such priming could influence both the timing of T-lineage commitment and the non-T-lineage potential retained by individual thymic progenitors. In the future, the LoxCode mouse model may facilitate the understanding of clonal relationships between multipotent DN subsets and non-T-cell populations in the thymus.

PU.1 deficiency significantly reduced thymic cellularity, consistent with its established role in maintaining thymocyte viability and proliferation before T-lineage commitment (14). Given the high expression of PU.1 in ETP and DN2a-1, this reduction likely reflects an impact on early DN substages. Interpretation of thymic phenotypes following systemic PU.1 deficiency is complicated by the established requirement of PU.1 in HSPC development, including lymphoid primed multipotent progenitors (LMPPs) and common lymphoid progenitors (CLPs) in the bone marrow (25, 49). To minimize this contribution of upstream defects, thymic analysis was performed one week following 4-OHT administration. Previous studies have shown that while CLPs begin to seed the thymus one week post adoptive transfer, thymic differentiation from less mature HSPCs requires longer timescales (53, 54). Therefore, the relatively short interval between 4-OHT administration and analysis was expected to limit the impact of impaired bone marrow hematopoiesis on thymic development. Consequently, the reductions observed in ETPs and DN2 thymocyte substages are most consistent with a direct requirement for PU.1 during early intrathymic development, although a contribution of altered prethymic progenitor supply cannot be completely excluded. Notably, expression of the HSPC-associated surface marker CD34 was also reduced on ETP and DN2 thymocytes in PU.1-deficient mice.

In contrast to PU.1, the role of the related ETS-family transcription factor SPI-B in T-cell development remains poorly defined. However, PU.1 and SPI-B exhibit partially compensatory roles in B-cell development (55); their expression in thymocytes is thought to be mutually exclusive (46, 56), reflecting PU.1-mediated repression of *Spib* (57). In line with previous studies, we found that *Spi1* expression was restricted to pre-commitment ETP and DN2a-1 stages, whereas *Spib* expression was upregulated only after T-lineage commitment in DN2b thymocytes (46, 56). Unexpectedly, however, *Spib*-tdTomato expression was highest in ETP and DN2a-1 cells. This may reflect activation of the *Spib* locus in thymus-seeding progenitors, although hematopoietic stem cells and CLPs have been reported to lack *Spib*-tdTomato expression (29). Consistent with previous studies (26), we found that SPI-B deficiency did not perturb T-cell development. Nevertheless, CD90 expression was reduced in SPI-B-deficient mice. CD90 itself also does not appear to be important for T-cell development (58), but as we now show, is a useful marker.

In summary, we demonstrate that the cell-surface markers CD90 and CD34 provide a higher-resolution framework for resolving DN2 thymocyte heterogeneity. This strategy distinguishes DN2a thymocytes into DN2a-1 and DN2a-2 substages, revealing that T-lineage commitment coincides closely with the transition between these populations. Although DN2a-2 thymocytes retain limited non-T lineage potential, they are predominantly T-lineage committed. Identifying these developmental intermediates enables more accurate mapping of the transcriptional, epigenetic, and clonal mechanisms underlying early T-cell specification and commitment.

## Data Availability

The datasets presented in this study can be found in online repositories. The names of the repository/repositories and accession number(s) can be found below: https://www.ncbi.nlm.nih.gov/, GSE336663.
