# Supplementary material for: Upregulation of CD90 correlates with T lineage commitment at the DN2a stage of T cell development
Source: Front Immunol. 2026 Sep 17;17:1940310. doi: 10.3389/fimmu.2026.1940310 (PMC13627384; doi:10.3389/fimmu.2026.1940310)
Supplement: Supplementary file 1 [file DataSheet1.pdf]

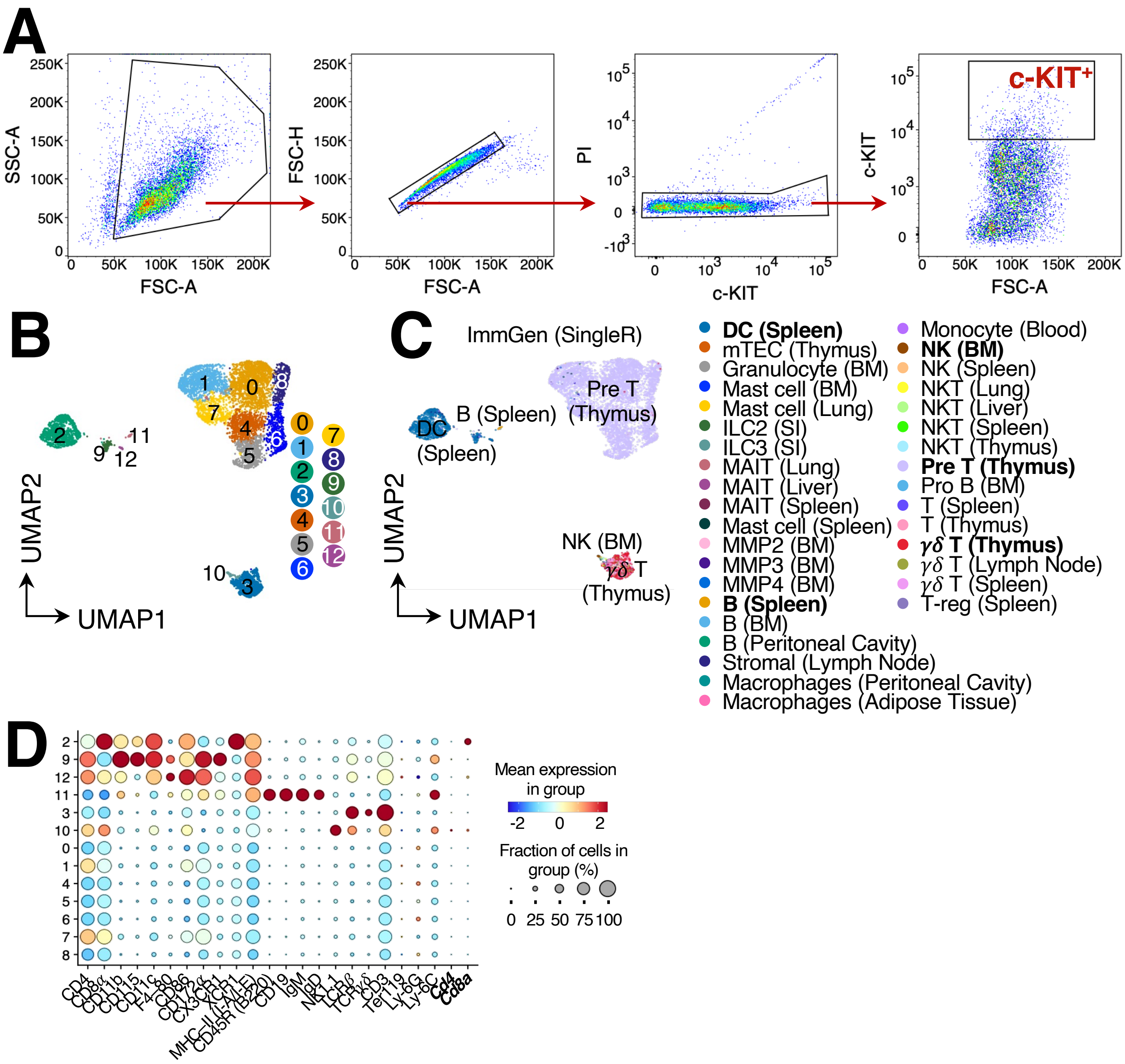

Supplementary Figure 1. CITE-seq analysis of c-KIT⁺ from the mouse thymus. (**A**) Total thymocytes from C57BL/6 mice were first depleted of CD4 and CD8-expressing cells with MACS magnetic beads, and then c-KIT⁺ cells were sorted for CITE-seq on the 10x Genomics platform. Shown is the FACS strategy. (**B**) WNN UMAP projection of the entire integrated (all three replicates) single-cell multiomic dataset (before exclusion of non-thymocytes), with the clusters identified using Seurat (resolution = 0.5). (**C**) Lineages (e.g., thymocytes, B cells, DC, etc) were annotated based on transcriptional similarity to the bulk RNA-seq datasets of immune cell populations from ImmGen using SingleR. (**D**) Expression of lineage-defining cell-surface proteins (detected by the CITE-seq), along with *Cd4* and *Cd8a* RNA, canonically used to define the major immune cell lineages in (C).

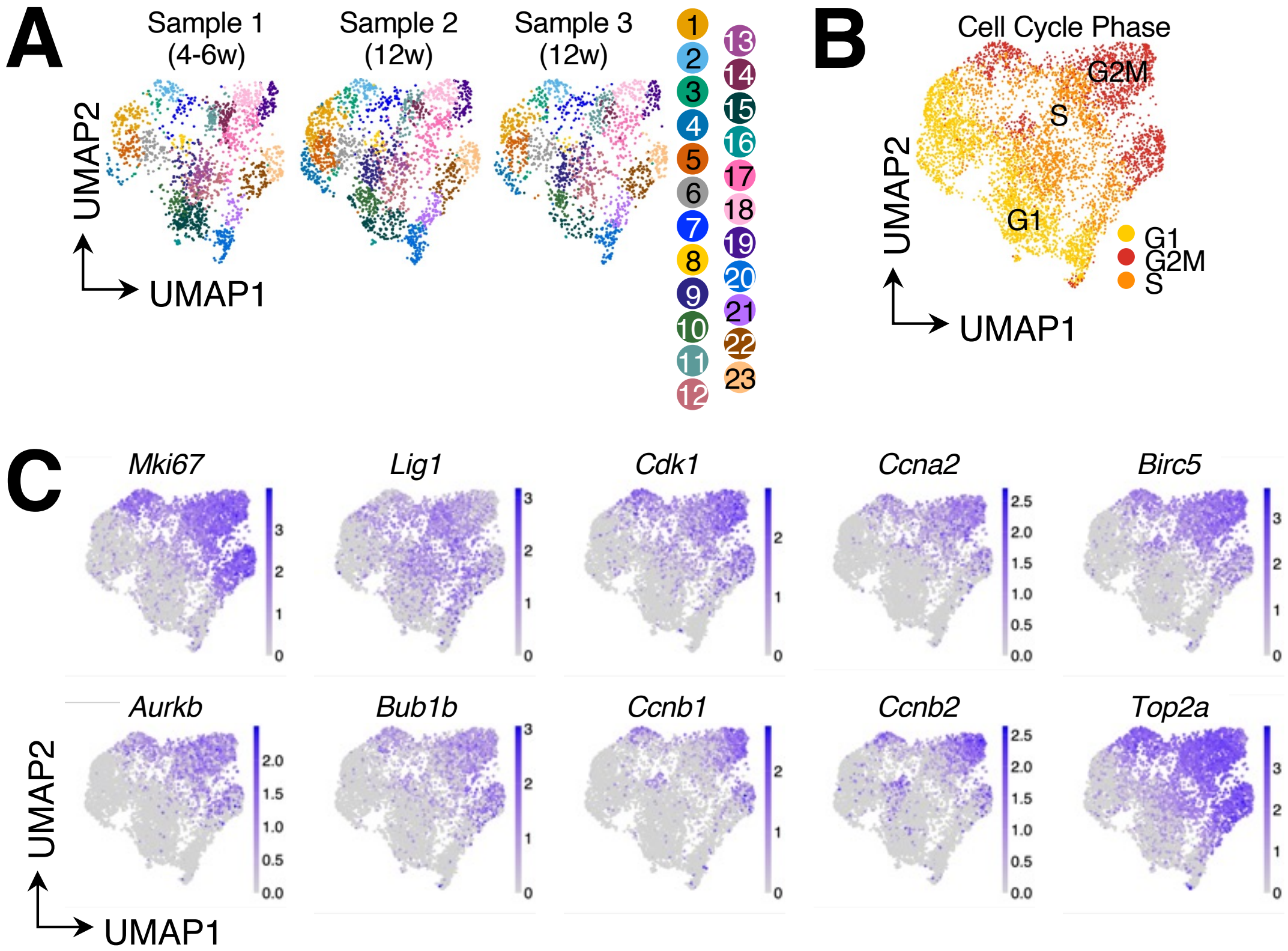

Supplementary Figure 2. Sample contribution and proliferative status within the CITE-seq dataset of ETP and DN2 thymocytes. The data include only c-KIT^{hi} CD4^{-} CD8^{-} DN thymocytes after all other lineages (e.g., B cells, DCs, etc.) were removed. (**A**) The contributions of each of the three independent experimental samples to the total UMAP projection in Figure 1A. (**B**) The total UMAP with cells color-coded by cell-cycle phase predicted by Seurat based on the expression of appropriate proliferation-associated genes. (**C**) Expression levels of genes associated with proliferation and cell cycle status.

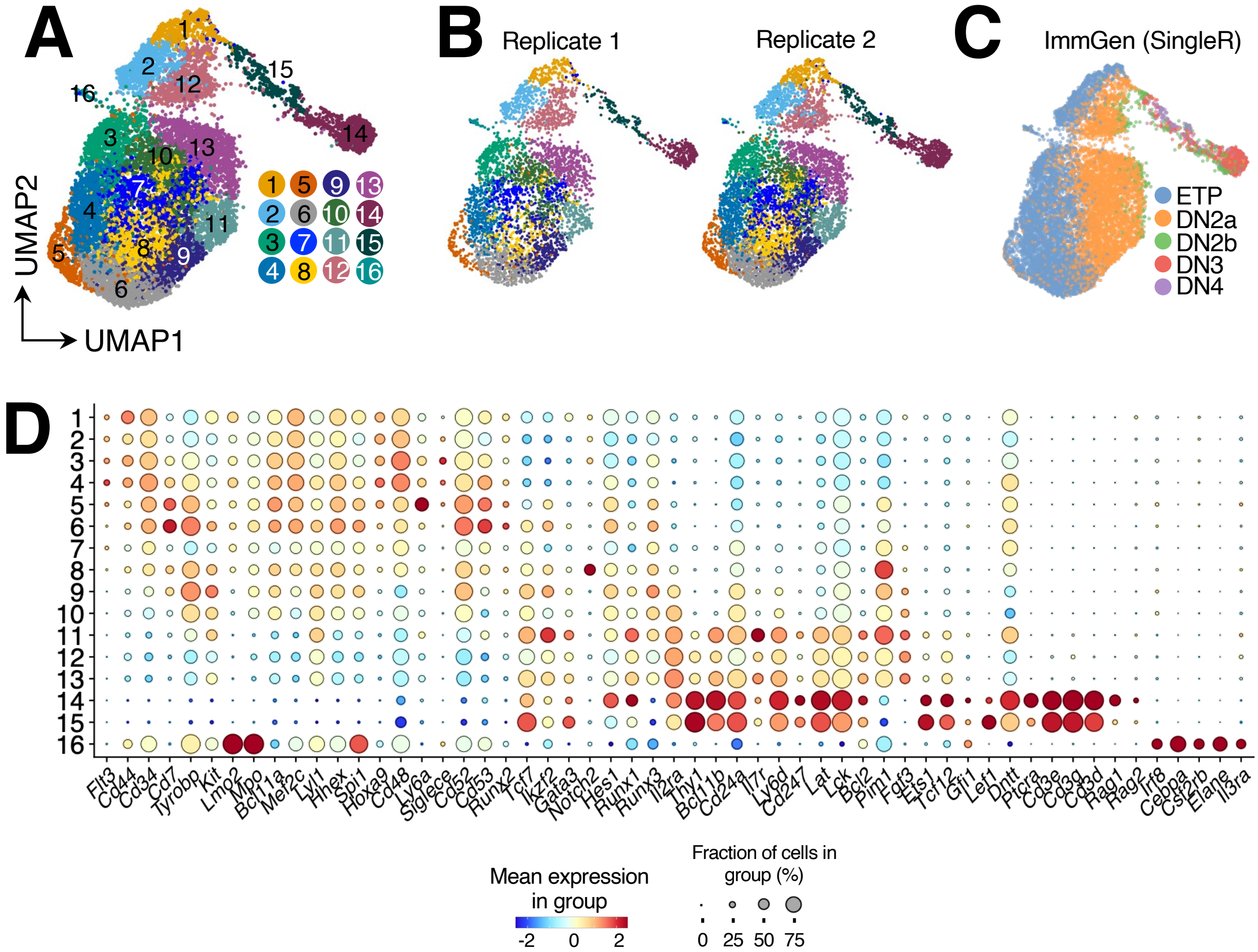

Supplementary Figure 3. Analysis of an additional scRNA-seq dataset of ETP to DN2a thymocytes. We analyzed two biological replicates of a previously published scRNA-seq dataset of ETP-to-DN2a thymocytes from C57BL/6 mice. (**A**) UMAP projection after merging the 2 replicates, with the clusters identified using Seurat (resolution = 1). The numbering reflects the approximate developmental progression. (**B**) Contributions of each replicate to the overall merged scRNA-seq dataset. (**C**) Cells were annotated based on transcriptional similarity to the bulk RNA-seq datasets of DN thymocytes from ImmGen using SingleR. (**D**) Dot plot showing expression of selected non-T and T specification genes involved in T-cell development.

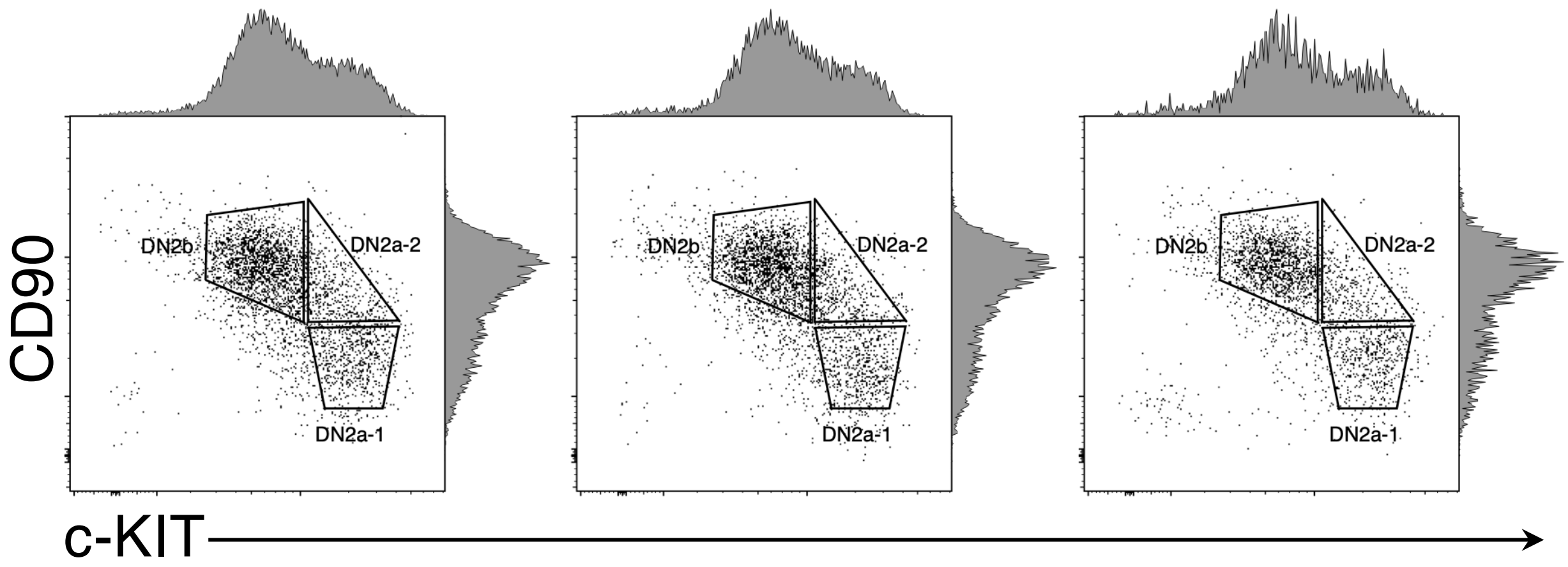

Supplementary Figure 4. Reproducible identification of CD90^{hi} versus CD90^{lo} DN2a thymocytes. Shown are representative flow cytometry plots from three independent samples, each derived from a different mouse and analyzed in separate experimental runs. The same predefined gate boundary was applied to all samples. Histograms adjacent to each plot show the fluorescence intensity distributions of c-KIT and CD90.

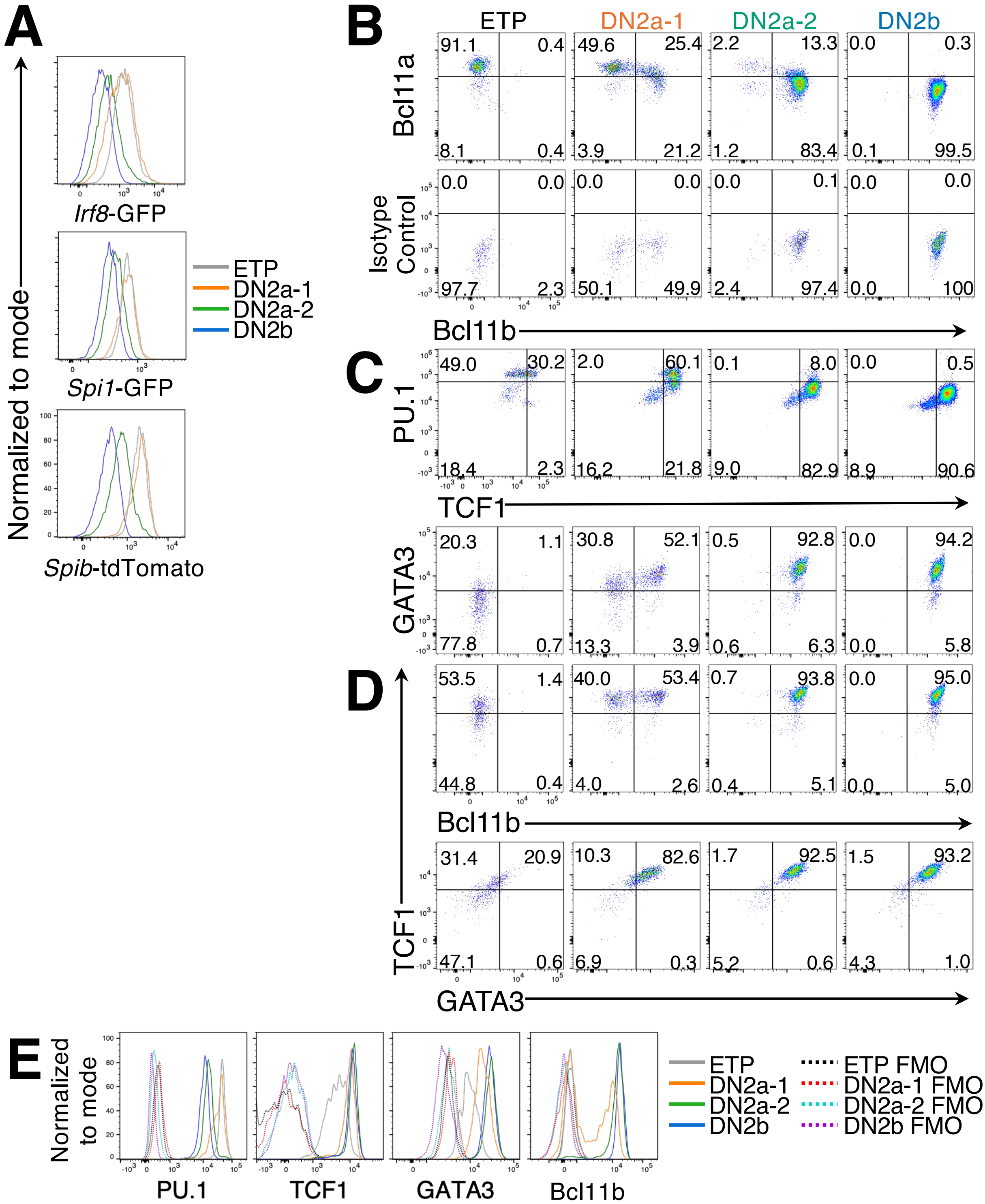

Supplementary Figure 5. Additional analysis of early thymocytes for expression of HSPC-associated transcription factors. (**A**) Representative flow cytometry histograms of ETPs and the DN2 subsets for *Irf8*-GFP, *Spi1*-GFP, or *Spib*-tdTomato expression in reporter mice. (**B**) Flow cytometry analysis of ETPs and DN2 subsets for Bcl11a and Bcl11b protein co-expression by intranuclear staining. A mouse IgG1 antibody was used as an isotype control for the Bcl11a antibody. (**C**) Flow cytometry analysis of ETPs and the DN2 subsets for PU.1 and TCF1 co-expression by intranuclear staining. **(D)** Flow cytometry analysis of ETPs and the DN2 subsets for GATA3, TCF1, and Bcl11b protein co-expression by intranuclear staining. (**E**) PU.1, TCF1, GATA3, and Bcl11b expression in ETP and DN2 subsets. Solid lines indicate marker staining and dotted lines indicate corresponding fluorescence minus one (FMO) controls.

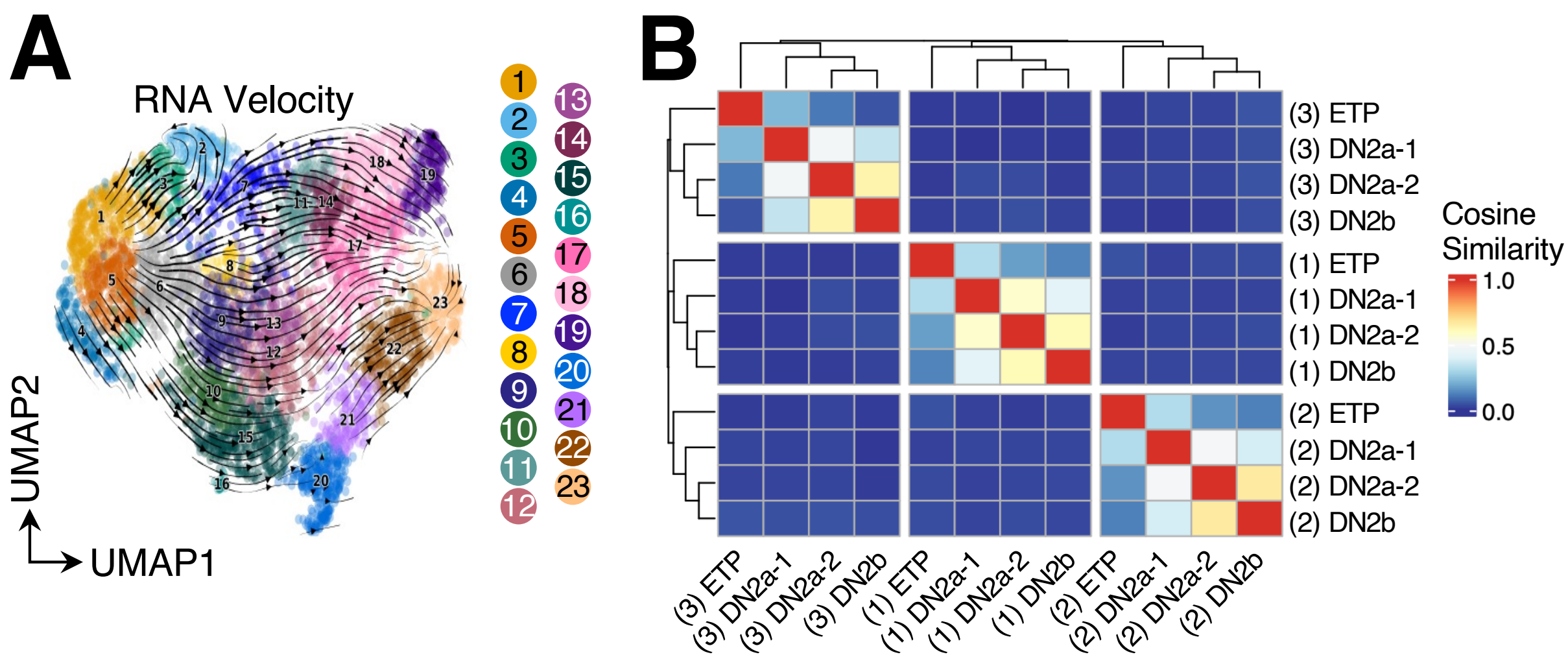

Supplementary Figure 6. Predicting the developmental relationships of ETP and the DN2 subsets based on computational inferences and *in vivo* clonal lineage tracing. (**A**) Prediction of developmental progression using RNA velocity analysis. Shown is the UMAP projection of the CITE-seq data, with cells color-coded by Seurat clusters and arrows representing the RNA velocities inferred from the ratio of unspliced to spliced transcripts. (**B**) LoxCode/*Rosa26*-CreER^{T2} mice were treated with 4-OHT to induce barcode recombination. 2 weeks later, ETP, DN2a-1, DN2a-2, and DN2b thymocytes were FACS-purified for barcode analysis from 3 individual mice. Shown is a hierarchically clustered heatmap for the cosine similarity values of detected barcodes between the indicated DN substages across the 3 biological replicates indicated by the numbers in brackets.

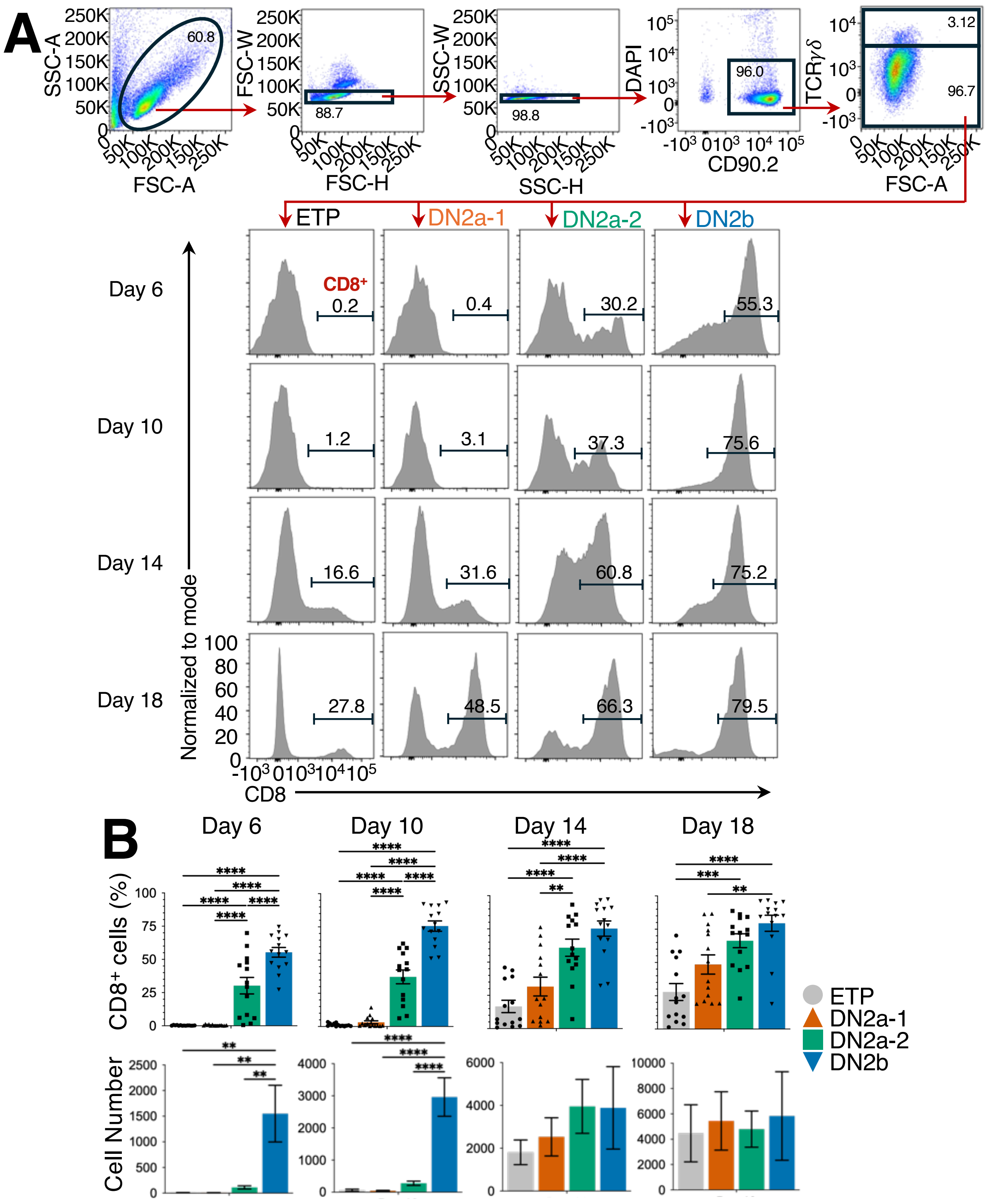

Supplementary Figure 7. Kinetics of αβ T-lineage differentiation derived from ETP and DN2 substages *in vitro*. ETPs or DN2 subsets were sorted from the thymus of C57BL/6 mice and co-cultured on OP9-DL1 stromal cells. The resulting cells were then analyzed by flow cytometry at the indicated time points. (**A**) αβ T-lineage differentiation was assessed by quantifying $CD8^+$ thymocytes, which identify $CD8^+TCR\beta^{lo}$ ISP and DP cells. A representative experiment is shown, with the top row representing the gating strategy applied to all replicates. (**B**) Cumulative data showing the mean ± SEM percentage (top) and number (bottom) of $CD8^+$ cells, with each dot representing an individual well from seven independent experimental repeats. One-way ANOVA with Tukey's post hoc test for multiple comparisons. *$P<0.05$, **$P<0.01$, ***$P<0.001$, ****$P<0.0001$.

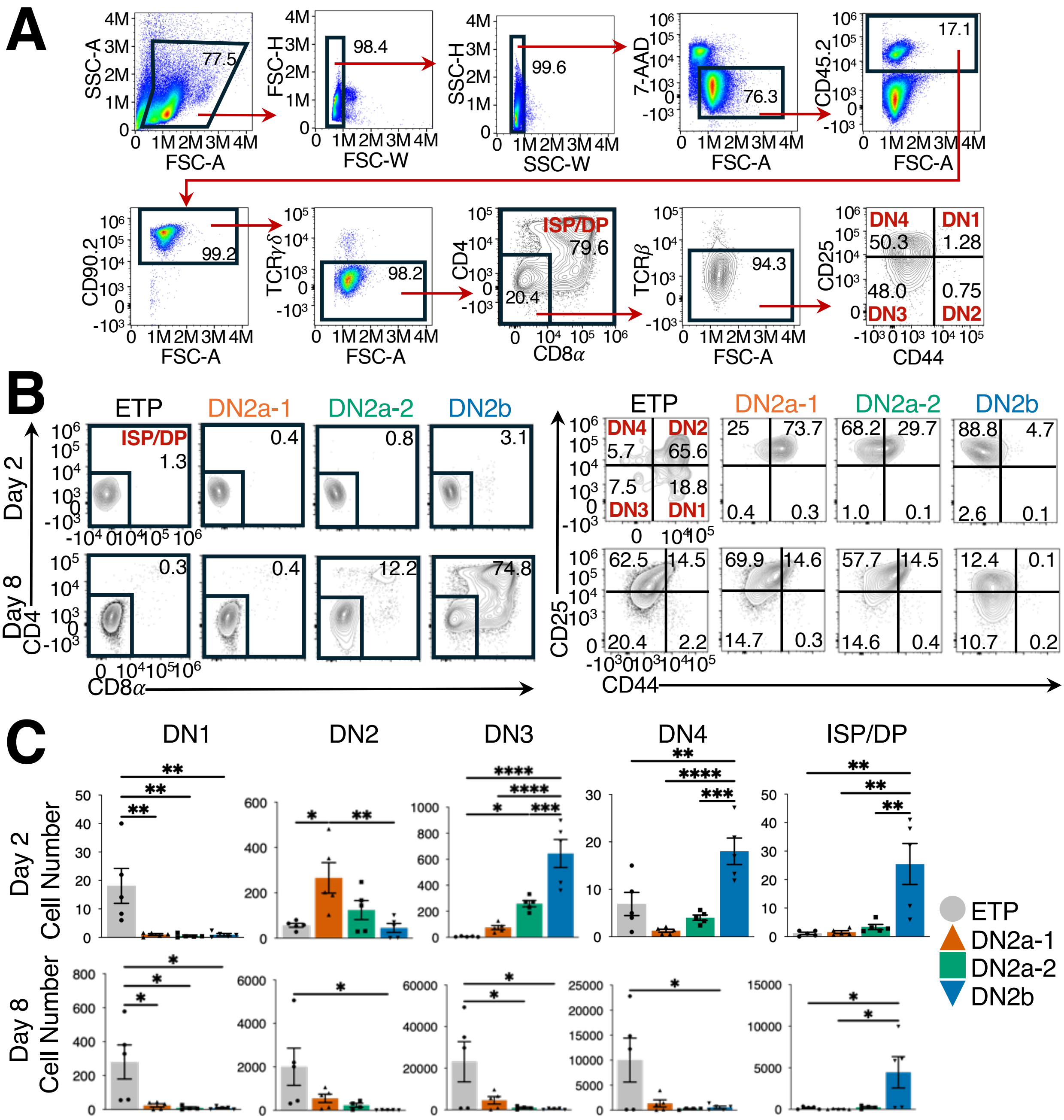

Supplementary Figure 8. Fine analysis of T-cell development in the OP9-DL1 cocultures. ETPs or DN2 substages were sorted from the thymus of C57BL/6 mice and co-cultured on OP9-DL1 stromal cells. The resulting progeny was analyzed by flow cytometry at the indicated time points. (**A**) Gating strategy used to analyze the resulting cells. Cells were gated on forward and side scatter parameters to exclude debris and doublets. Live cells were identified as 7-AAD$^-$. CD45$^+$CD90$^+$ thymocytes were identified, and TCRγδ$^+$ cells were excluded. ISP and DP thymocytes were defined as CD4$^+$ and/or CD8$^+$. DN thymocytes were defined as CD4$^-$CD8$^-$TCRβ$^-$ and further separated into DN1 (CD44$^+$CD25$^-$), DN2 (CD44$^+$CD25$^+$), DN3 (CD44$^-$CD25$^+$) and DN4 (CD44$^-$CD25$^-$). (**B**) Representative flow cytometry plots for CD4 versus CD8 expression (left) and CD44 versus CD25 expression within the DN gate (right). (**C**) Quantification of cell numbers detected in the OP9-DL1 cocultures. Data are presented as mean ± SEM, with each dot representing an individual well from two independent experimental repeats. One-way ANOVA with Tukey's post hoc test for multiple comparisons. *P<0.05, **P<0.01, ***P<0.001, ****P<0.0001.

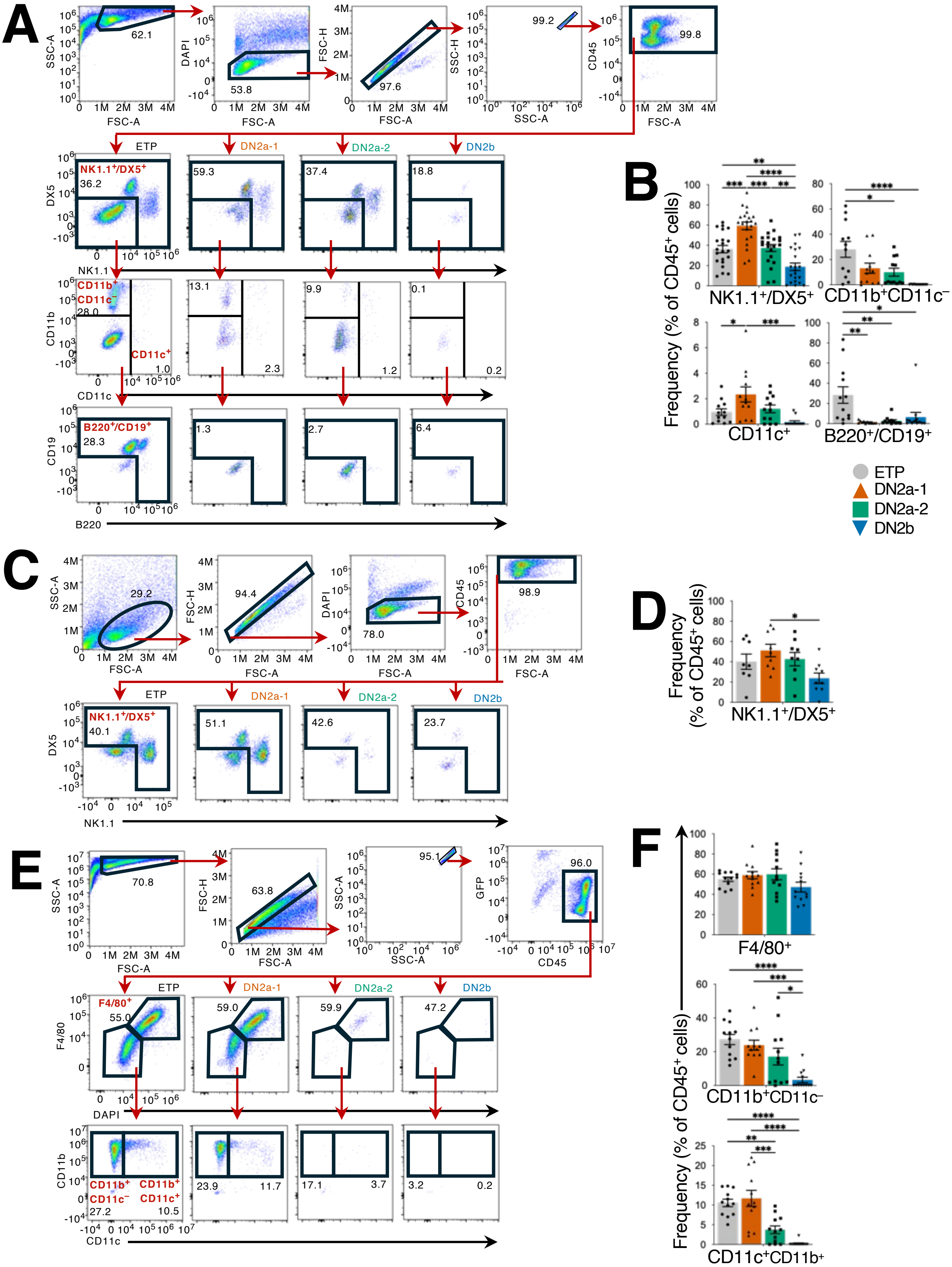
A
B
C
D
E
F
SSC-A
FSC-A
DAPI
FSC-H
SSC-H
CD45
62.1
53.8
97.6
99.2
99.8
ETP
DN2a-1
DN2a-2
DN2b
NK1.1+/DX5+
36.2
59.3
37.4
18.8
DX5
NK1.1
CD11b+
CD11c−
CD11c+
28.0
1.0
13.1
2.3
9.9
1.2
0.1
0.2
CD11b
CD11c
B220+/CD19+
28.3
1.3
2.7
6.4
CD19
B220
Frequency (% of CD45+ cells)
NK1.1+/DX5+
CD11b+CD11c−
CD11c+
B220+/CD19+
ETP
DN2a-1
DN2a-2
DN2b
29.2
94.4
78.0
98.9
40.1
51.1
42.6
23.7
Frequency
(% of CD45+ cells)
70.8
63.8
95.1
96.0
GFP
F4/80+
55.0
59.0
59.9
47.2
F4/80
27.2
10.5
23.9
11.7
17.1
3.7
3.2
0.2
F4/80+
CD11b+CD11c−
CD11c+CD11b+

Supplementary Figure 9. Analysis of non-T-lineage cells arising from ETP and DN2 substages *in vitro.* ETPs or DN2 substages were sorted from the thymus of C57BL/6 mice and co-cultured on OP9 stromal cells (without DL1) for 7 days under conditions that support differentiation into alternative lineages. (**A** and **B**) Representative flow cytometry plots and quantification of lineage outputs from ETP and DN2 substages cultured under non-T-lineage differentiation conditions (IL-7, Flt3L). Each dot represents an individual well from at least three independent experiments. In (**A**), the top row represents the gating strategy applied to all replicates. NK cells were identified as $NK1.1^+$ and/or $DX5^+$. The non-NK population was further separated into $CD11c^+$ DCs and $CD11b^+CD11c^-$ myeloid cells. We then analyzed $CD11b^-CD11c^-$ cells for B220 and CD19 expression to identify B cells. (**C** and **D**) Representative flow cytometry plots and quantification of lineage outputs from ETP and DN2 substages cultured under NK-lineage differentiation conditions (IL-7, Flt3L, IL-15). Each dot represents an individual well from at least three independent experimental repeats. In (**C**), the top row represents the gating strategy applied to all replicates. NK cells were identified as $NK1.1^+$ and/or $DX5^+$. (**E** and **F**) Representative flow cytometry plots and quantification of lineage outputs from ETP and DN2 substages cultured under myeloid-lineage differentiation conditions (IL-7, Flt3L, SCF, G-CSF, GM-CSF, IL-3). Each dot represents an individual well from at least four independent experimental repeats. In (**E**), the top row represents the gating strategy applied to all replicates. Macrophages were identified as $F4/80^+$. The non-macrophage population was further separated into $CD11b^+CD11c^+$ DCs and $CD11b^+CD11c^-$ myeloid cells. Data in (**B**), (**D**), and **(F)** are presented as mean ± SEM, with each dot representing an individual well from at least 3 independent experiments. Statistical significance was determined using one-way ANOVA with Tukey's post hoc test for multiple comparisons. $^*P<0.05$, $^{**}P<0.01$, $^{***}P<0.001$, $^{****}P<0.0001$.

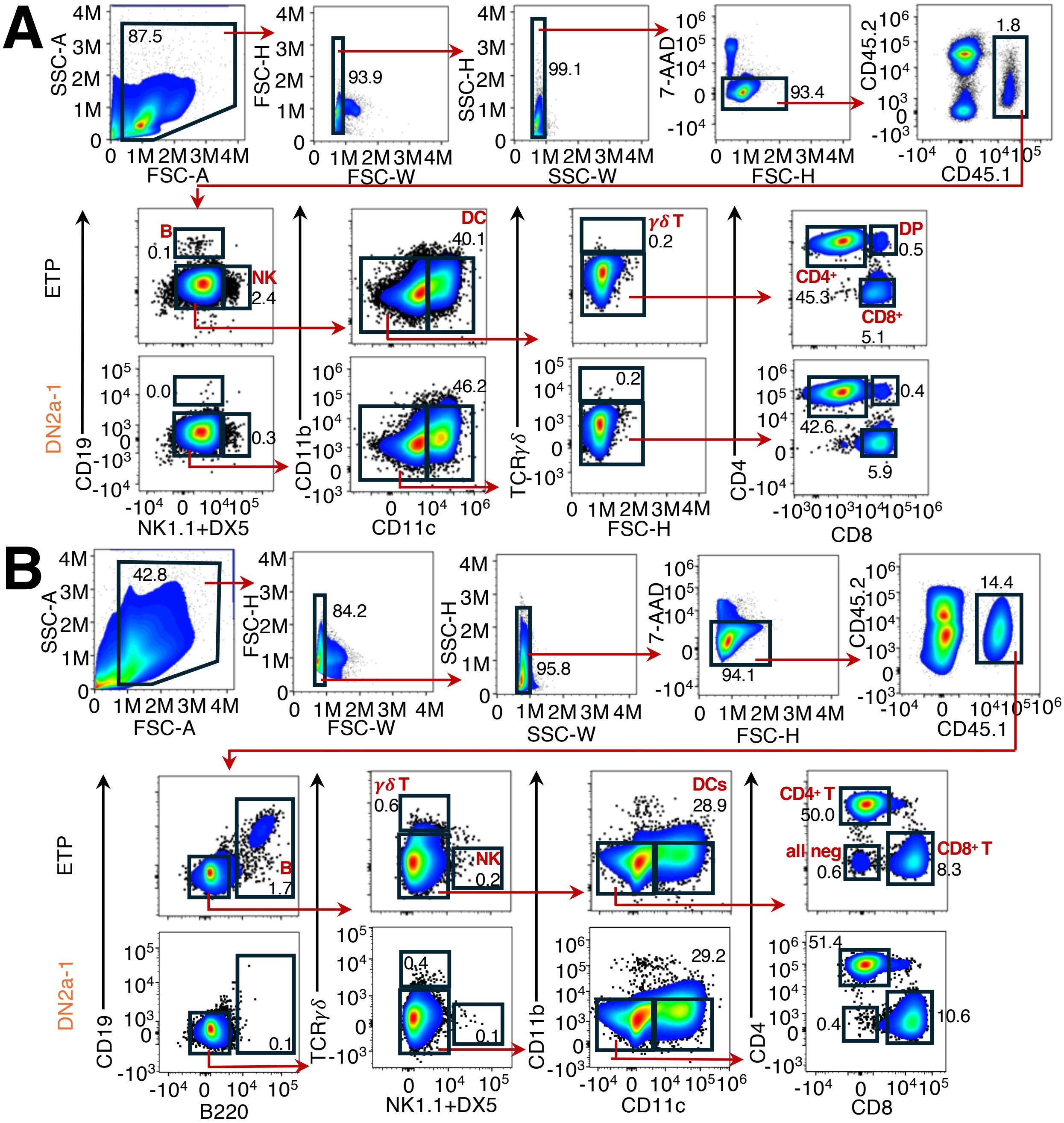

Supplementary Figure 10. Assessing the lineage potential of ETP and DN2a-1 cells *in vivo*. CD45.1^{+} ETPs or DN2a-1 thymocytes were adoptively transferred into sublethally irradiated *Rag1*$^{-/-}$ (CD45.2^{+}) mice. After 4 weeks, recipient tissues were analyzed by flow cytometry to assess the lineages derived from donor cells. (**A**) Representative analysis of the thymus. After excluding doublets, 7-AAD^{-} live donor-derived cells were identified as CD45.1^{+}. B cells were identified as CD19^{+}, and NK cells as NK1.1^{+}/DX5^{+}. Within the CD19^{-}NK1.1^{-}DX5^{-} population, DCs were identified as CD11c^{+}. Remaining CD11b^{-}CD11c^{-} cells were divided into CD4^{+} SP, CD8^{+} SP, or CD4^{+}CD8^{+} DP thymocytes. (**B**) Representative analysis of the spleen. After excluding doublets, 7-AAD^{-} live donor-derived cells were identified as CD45.1^{+}. B cells were identified as B220^{+}/CD19^{+}. Non-B cells were further separated into γδ T cells (TCRγδ^{+}) and NK cells (NK1.1^{+}/DX5^{+}). Within the TCRγδ^{-}NK1.1^{-}DX5^{-} population, DCs were identified as CD11c^{+}, while the CD11b^{-}CD11c^{-} cells were further subdivided into CD4^{+} or CD8^{+} T cells.

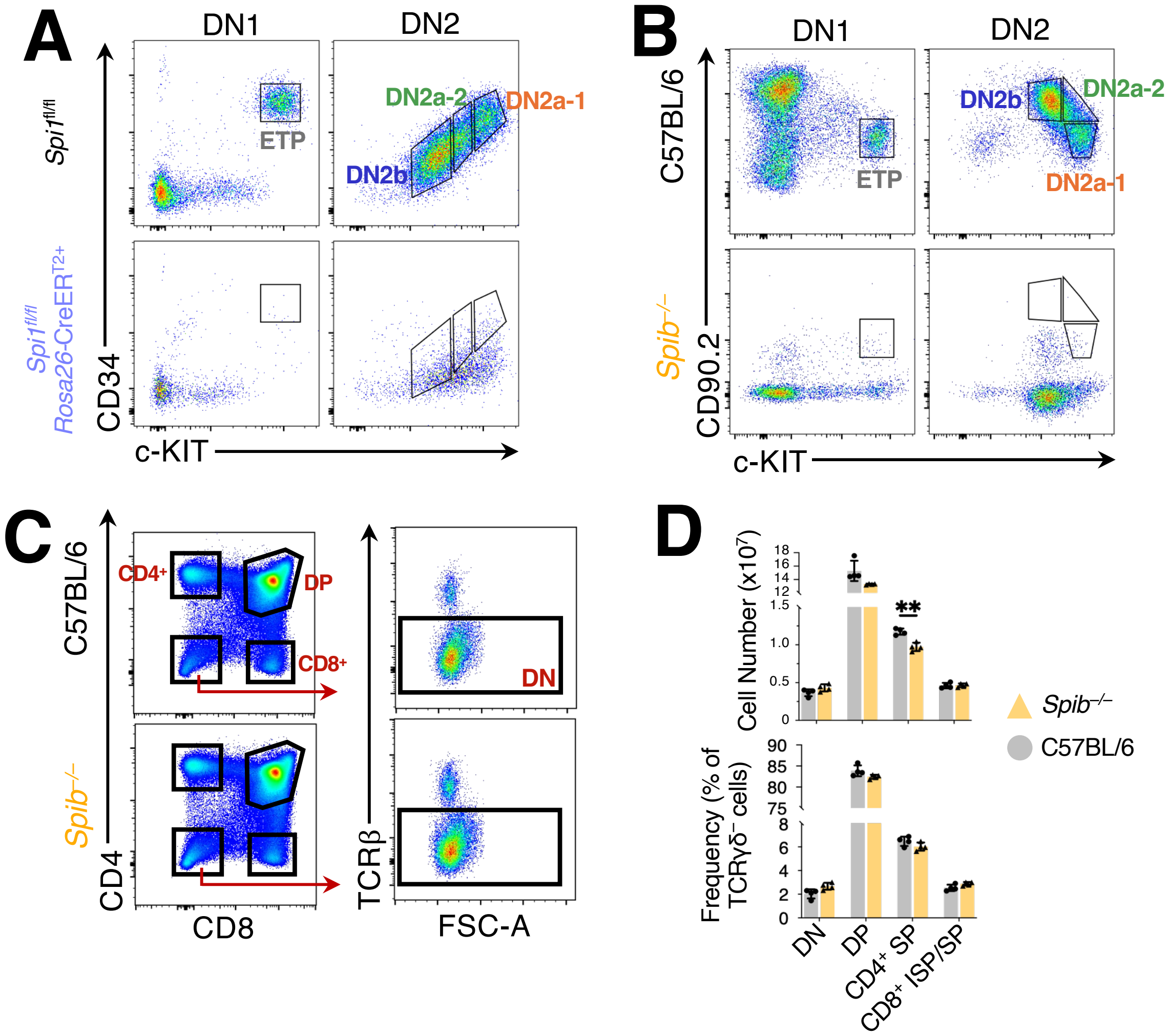

Supplementary Figure 11. Additional analyses of PU.1- and SPI-B-deficient mice. (**A**) *Spi1*$^{fl/fl}$ and *Spi1*$^{fl/fl}$*Rosa26*-CreER^{T2+} mice were injected with 4-OHT at 17 and 21 days of age, then the thymus was harvested at 24 days of age for analysis by flow cytometry. Shown is a representative analysis of selected DN1 (CD44^{+}CD25^{-}) (left) and DN2 (CD44^{+}CD25^{+}) (right) substages based on CD34 and c-KIT expression. (**B**) The thymi of *Spib*$^{-/-}$ and C57BL/6 wild-type mice were analyzed by flow cytometry. Shown is a representative analysis of selected DN1 (CD44^{+}CD25^{-}) (left) and DN2 (CD44^{+}CD25^{+}) (right) substages based on CD90 and c-KIT expression. (**C**) Shown is a representative analysis of CD4 and CD8 expression by total thymocytes. DN thymocytes are defined as CD4^{-}CD8^{-}TCRβ^{-}. (**D**) Analysis of individual animals for the number (top) and frequency (bottom) of each substage analyzed. The mean ± SD are shown, with each dot representing an individual mouse. Two-tailed unpaired Student's *t*-test with Holm-Šídák's multiple comparisons test. $^{**}P<0.01$.
